# *Rehmannia glutinosa* Replant Issues: Root Exudate-Rhizobiome Interactions Clearly Influence Replant Success

**DOI:** 10.3389/fmicb.2020.01413

**Published:** 2020-06-30

**Authors:** Bao Zhang, Leslie A. Weston, Mingjie Li, Xiaocheng Zhu, Paul A. Weston, Fajie Feng, Bingyong Zhang, Liuji Zhang, Li Gu, Zhongyi Zhang

**Affiliations:** ^1^Key Laboratory of Ministry of Education for Genetics, Breeding and Multiple Utilization of Crops, College of Agriculture, Fujian Agriculture and Forestry University, Fuzhou, China; ^2^Graham Centre for Agricultural Innovation, Charles Sturt University, Wagga Wagga, NSW, Australia; ^3^Henan Provincial People’s Hospital, Zhengzhou, China; ^4^Henan Province Chinese Medicine Research Institute, Zhengzhou, China

**Keywords:** allelopathy, pathogens, phytotoxicity, plant-soil feedbacks, replanting problems

## Abstract

Production of medicinal tubers of *Rehmannia glutinosa* is severely hindered by replanting issues. However, a mechanistic understanding of the plant-soil factors associated with replant problems is currently limited. Thus, we aimed to identify the *R. glutinosa* root exudates, evaluate their potential phytotoxicity and profile the interactions between the plant and its associated rhizobiome. Stereomicroscopy and liquid chromatography coupled to a quadrupole/time of flight mass spectrometer were used to monitor and identify secreted metabolites, respectively. Seedling bioassays were used to evaluate the phytotoxicity of *R. glutinosa* root exudates. Two complimentary experiments were performed to investigate allelochemical fate in rhizosphere soil and profile the associated microbiota. Root specific microbes were further isolated from *R. glutinosa* rhizosphere. Impacts of isolated strains were evaluated by co-cultivation on plate and on seedlings in tissue culture, with a focus on their pathogenicity. Interactions between key *R. glutinosa* root exudates and isolated rhizobiomes were investigated to understand the potential for plant-soil feedbacks. Quantification and phytotoxic analysis of metabolites released from *R. glutinosa* indicated catalpol was the most abundant and bioactive metabolite in root exudates. Subsequent microbial profiling in soil containing accumulated and ecologically significant levels of catalpol identified several taxa (e.g., *Agromyces*, *Lysobacter*, *Pseudomonas*, *Fusarium*) that were specifically shifted. Isolation of *R. glutinosa* rhizobiomes obtained several root specific strains. A significant antagonistic effect between strain Rh7 (*Pseudomonas aeruginosa*) and two pathogenic strains Rf1 (*Fusarium oxysporum*) and Rf2 (*Fusarium solani*) was observed. Notably, the growth of strain Rh7 and catalpol concentration showed a hormesis-like effect. Field investigation further indicated catalpol was increasingly accumulated in the rhizosphere of replanted *R. glutinosa*, suggesting that interactions of biocontrol agents and pathogens are likely regulated by the presence of bioactive root exudates and in turn impact the rhizo-ecological process. In summary, this research successfully monitored the release of *R. glutinosa* root exudates, identified several abundant bioactive *R. glutinosa* secreted metabolites, profiled associated root specific microbes, and investigated the plant-soil feedbacks potentially regulated by catalpol and associated rhizobiomes. Our findings provide new perspectives toward an enhanced understanding *R. glutinosa* replant problems.

## Introduction

The bioactive chemical constituents in herbal plants are typically specific to their native geographical areas (Su et al., 2005; Liu et al., 2015), and such geo-authentic regionality has been recommended as an important marker for establishment of improved quality standards for traditional Chinese medicines (Zhang B. et al., 2010; Yang et al., 2017). For example, *Rehmannia glutinosa* produced in Jiaozuo city, Henan province, China is accepted as one of the best traditional biomedicinal resources because of its high oligosaccharide, iridoid and phenylethanoid glycoside content (Wang et al., 2005; Qiu et al., 2010). However, replant problems, (the phenomena associated with the inhibition of germination, growth and yield of a plant when the same species or genus has been repeatedly cultivated in the same location) (Scotto la Massese, 1970; Bouhot, 1983; Yim et al., 2013), have severely limited the production of *R. glutinosa* (Zhang et al., 2013). Generally, field sites used for *R. glutinosa* cultivation require an 8–10 year period between replantation (Chen et al., 2007). However, due to the lack of arable land in Jiaozuo City, *R. glutinosa* is frequently replanted in the same field at intervals of less than 8 years, or cultivated beyond its geo-authentic region to meet market demand, resulting in a high incidence of disease, lower tuber yield, and poor product quality. Similar situations are also observed in other geo-authentic herbal plants (e.g., *Panax ginseng* and *Panax notoginseng*) (Yang et al., 2015; Dong et al., 2018a, b), which has severely hindered the production of medicinal products and sustainable development of traditional Chinese medicines (Gao et al., 2006; Zhang and Lin, 2009; Kong et al., 2011; Jiao et al., 2019). Given the serious replanting issues associated with production, *R. glutinosa* has been regarded as a model species for the study of replanting problems in perennial herbal plants (Zhang and Lin, 2009).

Numerous studies of related replanting issues have suggested that the deterioration of soil nutrient and other physico-chemical properties, infection by plant pathogens and pests, and phytotoxicity of associated metabolites released from plant residues and/or root exudates are implicated in reduced yield and quality (Westphal et al., 2002; Manici et al., 2013; Dong et al., 2018b). In recent research on *R. glutinosa*, organic matter and most available nutrients in replanted field sites were not impacted by years of replanting (Wu et al., 2013). However, the microbial communities in the rhizosphere of *R. glutinosa* in replanted field soil were dramatically shifted (Wu et al., 2013, 2016). Moreover, phytotoxic phenolic acids (e.g., ferulic acid and vanillic acid), which increasingly accumulated in the rhizosphere soil of *R. glutinosa* (Du et al., 2009a, b; Wu et al., 2009), were previously considered to be causal agents, as root exudates of *R. glutinosa* promoted mycelial growth, sporulation and toxin production of *Fusarium* spp. while inhibiting growth of the beneficial *Pseudomonas* strain (Wu et al., 2015). Bioassay showed that high concentration of phenolic acids were not only phytotoxic to *R. glutinosa* seedlings but also enhanced the oxidative damage and outbreak of wilting symptom in *R. glutinosa* together with *F. oxysporum* (Li et al., 2012, 2016). These findings suggested that extended monoculture of *R. glutinosa* altered the soil microbial community through the presence of phenolic acids in root exudates, resulting in the decline of beneficial bacteria with antagonistic activity against pathogens and the explosive growth and proliferation of *F. oxysporum*. Potentially, the phytotoxicity of accumulated phenolic acids together with the pathogenicity of proliferating associated phytopathogens were reported as potentially responsible for the replant problems/diseases of *R. glutinosa* (Wu et al., 2015, 2018a; Li et al., 2016).

However, this hypothesis fails to explain the phenomenon that *Zea mays* and *Triticum* spp., which frequently release similar levels of phenolic acids in field soils (Wu et al., 2000; Kidd et al., 2001) have not induced similar replant problems in subsequent *R. glutinosa* cultivation. Interestingly, when evaluating the previous methods utilized for phenolic acid quantification, we found that the authors extracted *R. glutinosa* rhizosphere soils under alkaline conditions (pH 13) and then acidified to pH 2.5 or 3, followed by re-extraction with ethyl acetate, redistillation, and redissolution in methanol for final identification and quantitation, resulting in the release of large quantities of phenolic acids that could be potentially artifactual (Solecka et al., 1999; Zhang and Gao, 2000). In fact, various metabolites released from root systems can regulate feedbacks of plant and soil microbes, serving as antimicrobials, signaling molecules, or microbial nutrients (Bais et al., 2008; Dong et al., 2018a; Kong et al., 2018). Once released, these compounds generally enter the plant rhizosphere and are subjected to physical sorption, chemical metal oxidation, and biological degradation (Huang et al., 1999; Inderjit., 2001). Apart from chemical breakdown, microbial degradation can limit the persistence of many secondary products of plant origin in the soil (Inderjit., 2005; Bais et al., 2008). Moreover, some root-released metabolites (e.g., aromatic esters, aromatic glycosides, and cinnamic acid derivatives) are unstable at high alkaline and/or acidic conditions. Here, we suggest that the previous methods employed for phenolic acid detection in the rhizosphere soil of *R. glutinosa* and subsequent investigation of interactions between phenolic acids and *R. glutinosa* rhizobiomes were potentially flawed. Thus, current studies to accurately identify metabolites in *R. glutinosa* root exudates are of critical importance to further evaluate its autotoxicity and the determine the rhizospheric ecological processes associated with replanting failure.

By employing metabolic profiling strategies, we successfully identified several phytotoxic metabolites including iridoid and phenylethanoid glycosides in the rhizosphere soil of *R. glutinosa* (Zhang et al., 2019). However, key questions remain, including whether such metabolites originated from *R. glutinosa* root exudation, the key constituents in *R. glutinosa* root exudates, and the potential interactions between key *R. glutinosa* root exudate and rhizobiomes. To address the above unanswered questions and further examine the mechanisms associated with *R. glutinosa* replant problems, we (1) monitored and identified the root exudates released from *R. glutinosa*; (2) assayed the phytotoxicity of the highly abundant metabolites in identified *R. glutinosa* root exudates; (3) deduced the location of release of the accumulated allelochemicals associated with exudation; (4) profiled and isolated the *R. glutinosa* root specific microbes; (5) assayed the pathogenicity and biocontrol efficiency of the isolated rhizosphere microbial strains; and (6) investigated the potential interactions between *R. glutinosa* and associated rhizobiomes mediated by key metabolites in root exudates under newly planted and replanted conditions.

## Materials and Methods

### Collection and Preparation of Plant and Soil Samples

To collect fresh root exudates, *R. glutinosa* seeds were germinated on double-layered filter paper moistened with 5 mL sterile water in sterile 9 cm plastic Petri dishes containing 80 seeds per dish at 26°C for 7 days in the dark, with three replicates and 10 dishes per replicate. All dishes were imaged using Leica M205 stereoscopic light microscopy (Leica microsystems, Weztlar, Germany) to view root hair exudation. Following harvest, seedling roots were immediately dipped into 20 mL methanol for less than 10 s to rapidly collect seedling root exudates (Supplementary Figures S1A,B). The root exudates were then dried under N_2_ gas at 35°C using dry block heater (Ratek DBH30, Boronia, VIC, Australia), and re-dissolved into 2 mL methanol for further analysis.

In natural field settings, *R. glutinosa* (cultivar “85-5”) was cultivated on the 28th May, 2017 using a row spacing of 25 cm and a spacing of 15 cm between plants in its geo-authentic region Wenxian city, Henan province, China (34°48′ N to 35°30′ N, 112°02′ E to 113° 38′ E). Rhizosphere soil of *R. glutinosa* and control soil in the neighboring field cultivated without *R. glutinosa* were collected across all key developmental stages of tuber growth including the beginning of tuber formation, the early stage of tuber expansion, middle stage of tuber expansion and formation of mature tubers at harvest at 90, 120, 150, and 180 days after seedling germination, respectively (Du et al., 2009a). Three plants were considered as one composite replicate from the field and collected without major disturbance, and six replicates were collected in total at each sampling. Root-zone soils were removed by softly shaking, and the soil closely attached to the roots was collected by brushing and sieved in a 2 mm mesh removing root hairs (Fujii et al., 2005). Samples consisting of 10 g of rhizosphere soil were dried at 60°C for 24 h to evaluate soil water content. The underground plant parts of *R. glutinosa* including tubers, were sampled at harvest on 23rd October, 2017 to study root exudation and bioaccumulation of metabolites in field soils cultivating *R. glutinosa*. The secondary roots, and the periderm, phloem and xylem tissues of tuber were carefully separated once collected (Supplementary Figures S1C,D). Additionally, the plants cultivated in various soil treatments including the newly planted soil (NP), the replanted soil for 1 year (RP1) and the replanted soil for 2 years (RP2) were collected to investigate survival rate and root dry weight of *R. glutinosa* under continuously monocultured conditions. Simultaneously, corresponding rhizosphere soils were also collected for quantification of the key *R. glutinosa* root exudate metabolite. All tissues including secondary roots, periderm, phloem and xylem were chopped and dried at 35°C for 3 h, and 3 g of each tissue was extracted with methanol (50 mL) under pressure (100 bar) at 35°C in two consecutive cycles. Extractions were evaporated to 10 mL at 35°C. Each soil sample (20 g) was immediately extracted with methanol (200 mL) for 1 h in sextuplicate using a shaker (IS-RDDS, Crystal, Dallas, TX, United States) at 170 revolutions/min. Soil extracts were concentrated to 10 mL at 40°C using a rotary evaporator (RE-6000A, Yarong, Shanghai, China). All the samples were filtered through a 0.22 μm polytetrafluoroethylene syringe filter and stored at -20°C before metabolic profiling.

### Metabolic Profiling of the Key Components in *R. glutinosa* Root Exudates and That Accumulated in Rhizosphere Soils

The root exudates and soil extracts were analyzed using a liquid chromatography (Agilent 1290, Santa Clara, CA, United States) coupled to a quadrupole/time of flight mass spectrometer (Agilent 6530, Agilent 1290, Santa Clara, CA, United States). A Poroshell 120 SB-C18 column (2.1 × 100 mm, particle size 2.7 μm) (Agilent, Santa Clara, CA, United States) was used for chromatographic separation at 35°C. The mobile phase consisted of A (0.1% formic acid in 95% acetonitrile) and B (0.1% formic acid in Milli-Q water). The same programs for gradient elution of molecules of interest and subsequent MS/MS analyses were used as described by Zhang et al. (2019). Metabolic profiling of the raw data was achieved using Agilent Molecular Structure Correlator (version B.08.00), Mass Profiler Professional (version 13.0), MassHunter Qualitative Analysis (version B.07.00) and MassHunter Profinder (Version B.08.00) (Agilent, Santa Clara, CA, United States). The entities present in all six samples replicates and specifically detected only in *R. glutinosa* root exudates and rhizosphere soils were identified/annotated by comparison of retention time, accurate masses, mass spectra and molecular features with our personal compound database and library (PCDL). The PCDL was built using Agilent PCDL manager ver. B.07.00 with known analytical standards based on our previous study (Zhang et al., 2019).

### Quantification of the Identified Metabolites in *R. glutinosa* Root Exudates, Rhizosphere Soils, and Different Tuber Tissues

The relative abundance of the six identified phenylethanoid glycosides (acteoside, isoacteoside, leucosceptoside A, 2′-actetylacetoside, martynoside, 2,4“Di-O-acetyl-3” ′-verbascoside) in *R. glutinosa* root exudates were analyzed using UHPLC-QTOF-MS with same gradient elution process as described above. The absolute concentration of candidate allelochemicals in the root exudates, rhizosphere soils and various tissues of *R. glutinosa* tubers were quantified via HPLC with UV detection. A Phenomenex SynergiTM Hydro-RP 80 Å column (4 μm, 250 × 4.6 mm) (Torrance, CA, United States) was used for separation of targeted metabolites. The mobile phase consisted of 0.2% phosphoric acid in Milli-Q water (solvent A) and acetonitrile (solvent B). The same gradient elution process described by Zhang et al. was used (Zhang et al., 2017), and calculate calibration curves were constructed using analytical standards (Supplementary Table S1).

### Phytotoxicity Assay of the Key Metabolites in *R. glutinosa* Root Exudates

Seeds of two sensitive and uniformly germinating bioindicator species, specifically *Lepidium sativum* L. (dicotyledon) and *Lolium perenne* L. (monocotyledon) were used in phytotoxicity evaluation and prepared for two different bioassays (Parker, 1966; Weston et al., 1989; Zhang et al., 2019). In Petri dish-filter bioassays, 0.5 mL of 0.125, 0.250, 0.500, 1.000, and 2.000 mg mL^–1^ of catalpol and acteoside solution was used to moisten double-layered filter paper in sterile 3.5 cm Petri dishes, with three replicates. Filter paper containing the same volume of sterilized water was used as the control. Ten seeds of *L. sativum* and *L. perenne* were transferred to filter paper and incubated at 25°C for 48 h and at 23°C for 72 h, respectively. A modified Parker soil assay was also performed in which both a non-pasteurized and pasteurized sand and soil mixture were packed in sterile square dishes and moistened with 20 mL of 1.250, 2.500, and 5.000 mg mL^–1^ of catalpol and acteoside solution, respectively (Zhang et al., 2019) to evaluate activity of each metabolite under pasteurized and non-pasteurized conditions in terms of soil microbiota. Fifteen *L. sativum* and *L. perenne* seeds were placed in each dish with three replications. The seeds were incubated at 25°C for 6 days in the dark. Percent inhibition of various concentrations of catalpol and acteoside on *L. sativum* and *L. perenne* radicle growth in the Petri-dish filter paper assay and modified Parker assay was calculated (Latif et al., 2020).

$\text{Inhibition}\text{percentage}\left(\%\right)=\frac{C\,K-\tau }{C\,K}\times 100\%$, CK and T were the average length of the radicle of seeds in controls and treatment, respectively.

### Complimentary Experiments

Catalpol proved to be the most abundant and phytotoxic metabolite in *R. glutinosa* root exudates in this study. Thus, two additional experiments were conducted to investigate the interactions of catalpol and associated soil microbes. Experiment 1 was performed to calculate the half-life (t_1__/__2_) of catalpol in soil in the following treatments: non-pasteurized and steam-pasteurized control soils planted without *R. glutinosa* were weighed (100 g) and transferred into sterile 9 cm plastic Petri dishes, with three replicate dishes evaluated for each sampling time. Soil pasteurization was achieved using the same method as described in modified Parker assay. The catalpol concentration in the soils was prepared to 10 μg g^–1^ (50% soil moisture content) by exogenous addition to soil and gentle shaking for uniform distribution. Dishes were placed in an incubator at 26°C in the dark. Twenty grams of soil was collected at 0, 6, 12, 24, 48, and 96 h after experimental initiation, with three replicates from each Petri dish per treatment, and immediately extracted with methanol to determine the relative abundance of catalpol in soil by UHPLC-QTOF-MS. Experiment 2 was performed to profile the specific microbes in catalpol accumulating soils as following: control soils planted without *R. glutinosa* were weighed (200 g) and transferred into 500 mL conical flasks, and sealed with four-layered gauze. Flasks were placed in an incubator at 26°C with humidity at 50% for 2 weeks to stabilize the soil microbial activities. After pre-incubation, the soil was moistened with 10 mL of 0.1 mg mL^–1^ catalpol solution on 2nd July 2018 and retreated with 5 mL of the same catalpol solution every 12 h (based on the half-life of catalpol in soil) for 60 days until 1st September 2018. Soils treated with the same volume of sterilized water were used as controls. Each treatment was replicated three times and all flasks were placed at 26°C with humidity at 50% during incubation. Two control soils were collected on 2nd July 2018 (CKT0) and 1st September 2018 (CKT1), and the soil treated with catalpol following the incubation period was collected on 1st September 2018, and is referred to as treatment C. Soil samples for DNA extraction were also collected at three respective time points as well.

### Microbial Profiling of Rhizobiome Responded to the Accumulation of Catalpol

Soil DNA was extracted using a PowerSoil DNA isolation kit (MoBio Laboratories, Carlsbad, CA, United States) following the manufacturer’s instructions and characterized by electrophoresis. DNA concentration was adjusted to 1 ng μL^–1^ with sterile water. Variable region 4 of bacterial 16S rRNA gene was selected and amplified with the specific primers 515F and 806R (Caporaso et al., 2012). Internal transcribed spacer (ITS1) amplification was performed using primers ITS5-1737F and ITS2-2043R (White et al., 1990). All PCR reactions were carried out in 30 μL with 15 μL of Phusion High-Fidelity PCR Master Mix (New England Biolabs, Ipswich, MA, United States), 0.2 μM of forward and reverse primers with 10 ng of template DNA. Thermal cycling consisted of initial denaturation at 98°C for 1 min, followed by 30 cycles of denaturation at 98°C for 10 s, annealing at 50°C for 30 s, elongation at 72°C for 30 s, and end with 72°C for 5 min. PCR products were then equally mixed and purified with Qiagen Gel Extraxtion Kit (Qiagen, Hilden, Germany) for parallel pyrosequencing. Libraries were generated using Illumina TruSeq DNA PCR-Free Library Preparation Kit, and assessed on the Qubit^@^ 2.0 Fluorometer (Thermo Fisher Scientific, San Jose, CA, United States) and Agilent Bioanalyzer 2100 system (Agilent Technologies, Santa Clara, CA, United States). The libraries were sequenced on an Illumina HiSeq2500 platform and 250 bp paired-end reads were generated. The paired-end reads were further assigned to each sample according to the unique barcodes and merged using FLASH (Tanja and Salzberg, 2011). Microbial sequences were analyzed using quantitative insights into microbial ecology software package (QIIME 2) (Caporaso et al., 2010). The effective sequences with greater than or equal to 97% similarity were assigned to the same operational taxonomic units (OTUs), and the taxonomic information for each representative sequence was annotated using the RDP classifier (Qiong et al., 2007). Alpha diversity including observed species, Chao 1, ACE, and Shannon indices were analyzed to investigate the richness and species diversity within samples, and weighted Unifrac distance was calculated and used for principal coordinate analysis (PCoA) and unweighted pair group method with arithmetic mean clustering (UPGMA) (Chao, 1984; Ludwig and Reynolds, 1988; Joseph et al., 2015). The PICRUSt v.1.1.2 and FUNGuild v1.1 were used to predict the bacterial and fungal functional composition (Langille et al., 2013; Nguyen et al., 2016), respectively.

### Isolation of *R. glutinosa* Root Specific Microbes and Their Pathogenic and Biocontrol Potentials Assay

Rhizosphere soils of newly planted (NP) and replanted *R. glutinosa* for 2 years (RP2) were collected and immediately homogenized (10 g) in 100 mL sterile water for isolation of root specific microbes. The soil suspension was subjected to 10-fold serial dilution, and 100 μL of each diluent (10^–2^ to 10^–5^) was transferred to Petri dishes with Luria-Bertani medium (LB) and potato dextrose agar medium (PDA) with 0.2 g L^–1^ chloramphenicol. The LB and PDA plates were incubated at 36°C and 26°C for 3 days for bacteria and fungi isolation, respectively. All bacterial colonies and fungal hyphae were selected and purified for further identification. Only the isolated strains showing close genetic relationship with those that were specifically profiled in the soil with accumulated catalpol were selected for assessment of their pathogenicity and biocontrol efficacy. Of these, three strains identified as *Pseudomonas* aeruginosa (strain Rh7), *Fusarium oxysporum* (strain Rf1), and *Fusarium* solani (strain Rf2) were screened and cultivated. The bacterial strain Rh7 and fungal strains Rf1 and Rf2 were cultivated for 12 h in LB medium at 36°C with shaking at 120 rpm and for 24 h in PDA broth medium at 26°C with shaking at 150 rpm, respectively. The aliquots were used for further assays.

To investigate effects of key *R. glutinosa* root exudates on growth of root specific microbes, bacterial aliquot (200 μL) was inoculated into 50 mL of LB broth medium containing different dosages of catalpol (10, 20, 40, and 80 μg mL^–1^). The growth of strain Rh7 was determined by measuring the OD_600_ monitored spectrophotometrically at 4, 8, 12, 16, 20, and 24 h. Soil-extracted broth and agar medium were prepared using the supernatant of water extract of control soil and adjusted catalpol concentrations to 10, 20, 40, and 80 μg mL^–1^ according to the method described by Wu et al. (2015). Fungal aliquots (5 μL) were inoculated onto the center of 9 cm diameter Petri dish filled with a 10-fold dilution of soil-extracted agar medium containing different dosages of catalpol and incubated at 26°C for later assessment of the colony diameter after 8 days. Fungal aliquots (200 μL) were inoculated into 50 mL of soil-extracted broth medium containing same gradients of catalpol and incubated at 26°C for 8 days with shaking at 150 rpm. The number of conidia was calculated by counting of spores using a hemocytometer (Wu et al., 2015). Additionally, the potential interactions between strain Rh7 and two strain Rf1 and Rf2 were assayed by co-cultivation on plate and on *R. glutinosa* tissue culture seedlings. For antagonism assay, 5 μL of fungal aliquot was transferred to the center of PDA plates, and strain Rh7 was inoculated at the periphery (Supplementary Figures S2A,B). Plates were incubated at 26°C for 6 days to recorded the antagonistic activity of Rh7 against Rf1 and Rf2. For potential biocontrol assay of strain Rh7, the roots of 30 days-old *R. glutinosa* seedlings (from tissue culture) were inoculated with fungal hyphae, and specifically 20 μL of aliquot of strain Rh7 was inoculated 1 cm away from the seedling roots. The growth status of seedlings was recorded after 12 days (Supplementary Figure S3). Three repetitions were performed for the above-mentioned experimental settings.

### Statistical Analysis

The difference in abundance of catalpol in various tuber tissues of *R. glutinosa* and rhizosphere soils, and the differences of microbial alpha diversity between soil sample groups were analyzed using standard *T*-tests (*p* < 0.05) by the statistical software SPSS 20.0. Each value was expressed as the mean of replicates ± standard deviation. The statistical analysis of microbial beta diversity was achieved using MetaStat and liner discriminant analysis effect size (LEfSe, LDA score > 4.0) methods (White et al., 2009; Segata et al., 2011). The heatmaps of predicted microbial functions were produced using R software version 3.5.1 combined with pheatmap v1.0.12 package.

## Results

### Identification of Metabolites in *R. glutinosa* Root Exudates

**FIGURE 1:**
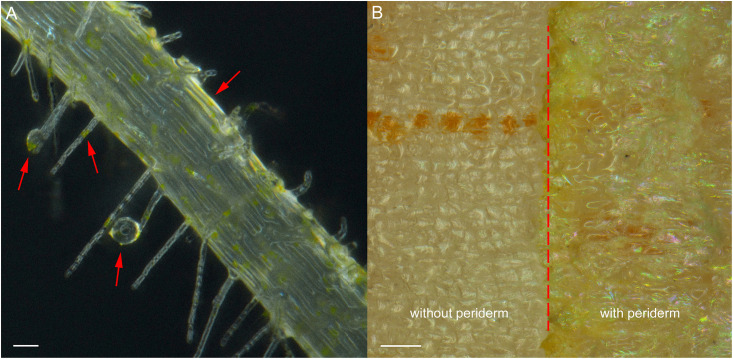
Root exudates and colored epidermal tissues in seedling roots and mature tuber periderm of *R. glutinosa*. **(A)** Bright field image of root hairs of 7-day-old *R. glutinosa* seedling, showing releasing of yellow exudates. Bar, 50 μm. **(B)** Bright field image of tuber surface of 180-day-old *R. glutinosa*, showing accumulation of yellow metabolites in the periderm (with periderm) and in cortex (without periderm). Bar, 0.5 cm.

**FIGURE 2:**
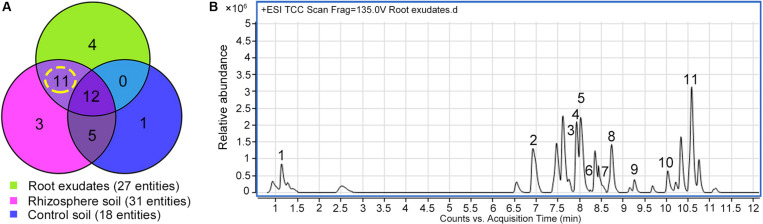
Screening of the key metabolites in *R. glutinosa* root exudates which subsequently accumulated in its rhizosphere. **(A)** Venn diagram of metabolite distribution in root exudates of 7-day-old *R. glutinosa* seedlings and rhizosphere soil of 180-day-old *R. glutinosa*. **(B)** Typical total extracted compound chromatogram (TCC) of *R. glutinosa* root exudates at 7 days of age.

Exudation was clearly evident in *R. glutinosa* seedlings as noted by the considerable quantities of yellow-colored droplets observed accumulating at the tips and within the periderm of living roots (Figure 1A). Yellow colored constituents also accumulated in the periderm of mature tubers of *R. glutinosa* (Figure 1B). To profile the key secondary products released from *R. glutinosa* roots and those accumulating in the *R. glutinosa* rhizosphere in natural field settings, seedling root exudates and rhizosphere soils collected at harvest (180 days) were profiled and analyzed, and are presented in Figure 2. By direct comparison with the soil cultivated without *R. glutinosa* (control soil), we found that eleven metabolites specific to *R. glutinosa* root exudates and rhizosphere soil were present. By searching against our PCDL, one iridoid glycoside (catalpol) and six phenylethanoid glycosides including acteoside, isoacteoside, leucosceptoside A, 2′-actetylacetoside, martynoside and 2,4“Di-O-acetyl-3” ′-verbascoside were positively identified. Compounds 3 and 4 were tentatively annotated as rehmaionoside A/B and rehmaionoside C. Due to the lack of standards, further bioassay and quantification of compounds 3 and 4 have not yet been performed at this time. No detailed structural information was obtained for compounds 2 and 11. Thus, their structures remain currently unknown. The identified/annotated key metabolites in root exudates accumulating in the *R. glutinosa* soil rhizosphere are summarized in Supplementary Table S2.

### Quantification of the Key Metabolites in *R. glutinosa* Root Exudates and Rhizosphere Soils

**FIGURE 3:**
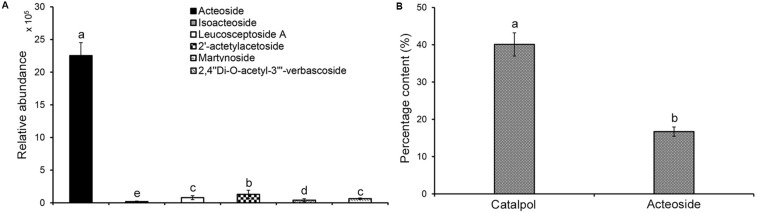
Quantification of catalpol and six phenylethanoid glycosides in *R. glutinosa* root exudates. **(A)** Relative abundance of six phenylethanoid glycosides in *R. glutinosa* root exudates. **(B)** Percentage content of catalpol and acteoside in *R. glutinosa* root exudates. Different letters indicate statistical difference (*p* < 0.05).

Analysis of the relative abundance of acteoside, isoacteoside, leucosceptoside A, 2′-actetylacetoside, martynoside, 2,4“Di-O-acetyl-3” ′-verbascoside in *R. glutinosa* root exudates revealed that acteoside was the most abundant phenylethanoid glycoside (Figure 3A). Previous analyses also determined that the relative abundance of acteoside was higher in *R. glutinosa* rhizosphere than other related phenylethanoid glycosides (Zhang et al., 2019). HPLC analysis of the iridoid glycoside (catalpol) and the most abundant phenylethanoid glycoside (acteoside) indicated that catalpol accounted for a much higher percentage (40.1%) by weight than that of acteoside (16.7%) in the crude *R. glutinosa* root exudates (Figure 3B), suggesting catalpol and acteoside were the two most abundant metabolites in *R. glutinosa* root exudates.

**FIGURE 4:**
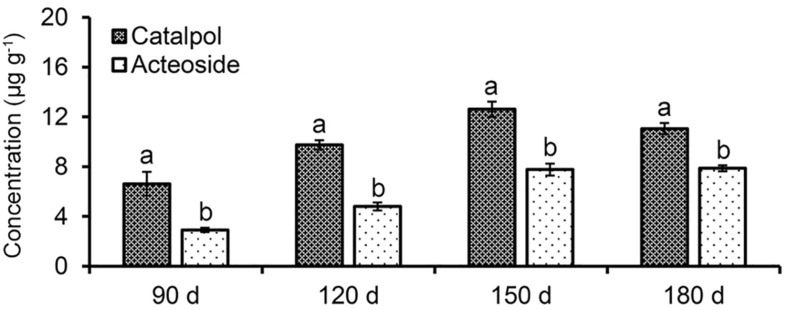
Quantification of catalpol and acteoside in rhizosphere soils at various growth stages of *R. glutinosa*. Four *R. glutinosa* growth stages including the beginning of tuber formation, the early stage of tuber expansion, middle stage of tuber expansion and mature tubers at harvest were samples at 90, 120, 150, and 180 days after seedling germination, respectively. Different letters indicate statistical difference (*p* < 0.05).

Absolute quantification of catalpol and acteoside in rhizosphere soils collected at different *R. glutinosa* growth stages showed that these two metabolites accumulated at increasing levels in *R. glutinosa* rhizosphere with tuber development. Moreover, significantly higher concentrations of catalpol were detected in rhizosphere of *R. glutinosa* at each successive growth stage. The rhizosphere soil collected at middle-stage of tuber expansion (150 days) contained the highest catalpol concentration (12.6 μg g^–1^, 51.1% moisture content), followed by the harvest stage (180 days) (11.1 μg g^–1^, 49.3% moisture content). A higher acteoside concentration was also found in the rhizosphere soils collected at harvest stage (7.87 μg g^–1^) and the middle-stage of tuber expansion (7.77 μg g^–1^) (Figure 4 and Supplementary Figure S4).

### Distribution of Targeted Metabolites in Different Tissues of *R. glutinosa* Tubers

**FIGURE 5:**
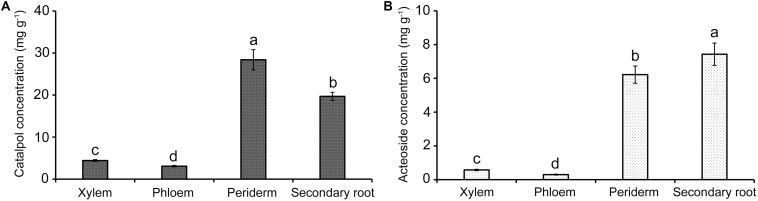
Quantification of catalpol **(A)** and acteoside **(B)** in various tissues of *R. glutinosa* tubers. Different letters indicate statistical difference (*p* < 0.05).

HPLC quantification showed that catalpol and acteoside were present in high concentrations in two tissues, the tuber periderm and secondary roots of *R. glutinosa*. The highest catalpol concentration was detected in the periderm (28.41 mg g^–1^), followed by secondary roots (19.70 mg g^–1^). The greatest concentration of acteoside was also observed in the secondary roots (7.43 mg g^–1^), while the concentration of acteoside in the periderm also reached 6.22 mg g^–1^. However, the abundance of catalpol in the xylem and phloem was significantly lower at less than 5.00 mg g^–1^. In contrast, the acteoside concentration in xylem and phloem was greatly reduced at 0.57 and 0.30 mg g^–1^, respectively (Figure 5). Together with the finding of these two compounds in the soil samples, the release of these metabolites probably occurred at the periderm and secondary roots through exudation or decomposition of tissues over time.

### Phytotoxicity Assay of Catalpol and Acteoside

**FIGURE 6:**
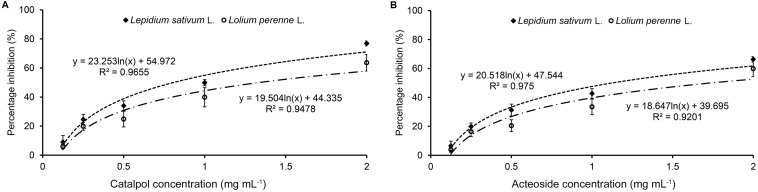
Petri dish-filter paper phytotoxicity assay of catalpol **(A)** and acteoside **(B)** on *L. sativum* (dicotyledon) and *L. perenne* (monocotyledon).

**FIGURE 7:**
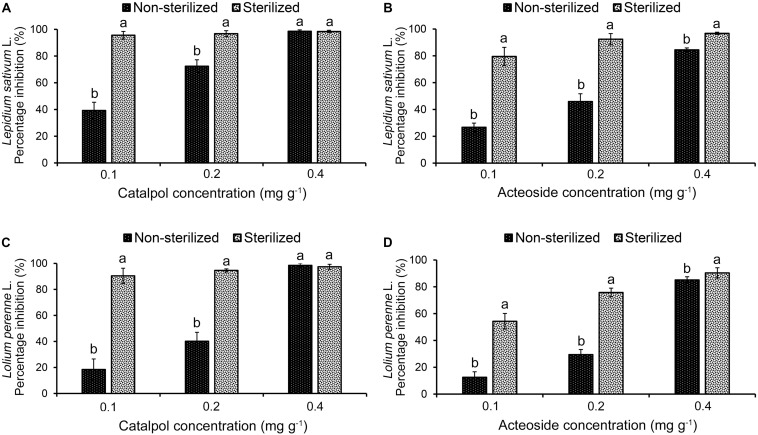
Modified Parker phytotoxicity assay to assess the activity of catalpol and acteoside in both pasteurized and non-pasteurized soils on *L. sativum* (dicotyledon) and *L. perenne* (monocotyledon). **(A)** Percentage inhibition of catalpol on radicle growth of *L. sativum*. **(B)** Percentage inhibition of acteoside on radicle growth of *L. sativum*. **(C)** Percentage inhibition of catalpol on radicle growth of *L. perenne*. **(D)** Percentage inhibition of acteoside on radicle growth of *L. perenne*. Different letters indicate statistical difference (*p* < 0.05).

Results of Petri dish-filter paper bioassay indicated that the percentage inhibition of radicle growth of *L. sativum* and *L. perenne* logarithmically increased with increasing concentration of catalpol and acteoside (Figure 6). The calculated IC_50_ values revealed that the phytotoxic or potential allelopathic activity of catalpol on *L. sativum* (0.81 mg mL^–1^) was greater than that on *L. perenne* (1.34 mg mL^–1^). By contrast, the phytotoxicity of acteoside on *L. sativum* (IC_50_ = 1.13 mg mL^–1^) and *L. perenne* (IC_50_ = 1.74 mg mL^–1^) were less than that of catalpol. In the modified Parker assay, the catalpol and acteoside progressively inhibited radicle growth of *L. sativum* and *L. perenne* with increasing concentrations (Figure 7). The percentage inhibition of the radicle growth of these two species by catalpol at 0.4 mg g^–1^ in both pasteurized and non-pasteurized media reached 95%. In contrast to catalpol, acteoside showed relatively less activity on radicle growth of *L. sativum* and *L. perenne* at each concentration. Both plant growth bioassays indicated that catalpol had greater than phytotoxicity did acteoside. Interestingly, the phytotoxicity of catalpol and acteoside were both significantly higher in the pasteurized soil media than that in non-pasteurized media at all concentrations (0.1, 0.2, and 0.4 mg g^–1^), suggesting their allelopathic efficacy and fate are potentially impacted by soil microbes. As catalpol was the most abundant metabolite identified in the *R. glutinosa* rhizosphere and was significantly more active than acteoside in this study, we focused our efforts on catalpol in further studies examining the potential plant-soil chemical feedback mediated by *R. glutinosa* root exudates. The role of acteoside and other phenylethanoid glycosides in the plant-soil system will be evaluated.

### Biodegradation Dynamics of Catalpol in the Soil

**FIGURE 8:**
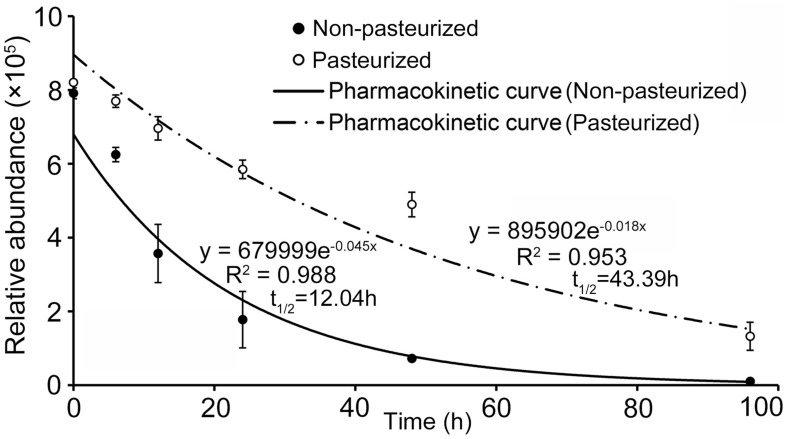
Degradation and soil half-life of catalpol in non-pasteurized and pasteurized soils.

UHPLC-QTOF-MS analysis showed that the concentration of catalpol in both non-pasteurized and pasteurized soil exhibited an exponential decrease over with time, and the pharmacokinetic degradation curves were generated as *y* = 679999*e*^–0.045^*^*x*^* and *y* = 895902*e*^–0.018^*^*x*^*, respectively (Figure 8). The calculated half-life revealed that catalpol degraded more rapidly in non-pasteurized soil (t_1__/__2_ = 12.04 h) than that in pasteurized soil (t_1__/__2_ = 43.39 h), further suggesting soil microbial involvement in the biodegradation of catalpol.

### The Impacts of Accumulated Catalpol on Rhizospheric Microbial Composition

The structures of microbial community in soils accumulated with (C) and without (CKT0 and CKT1) catalpol were accessed using deep pyrosequencing amplicons. A total of 730 250 and 610 220 effective tags with annotation of bacterial and fungal species were obtained from 12 soil samples, respectively. Singletons of bacteria and fungus, which were removed from the dataset before further analysis, accounted for only 2.25% (16 467 tags) and 2.10% (12 842 tags) of the total tags (Supplementary Figure S5). All created libraries created representing the bacterial and fungal communities as well as the rarefaction curves were approaching plateaus with the goods coverages at 0.992 (Supplementary Figure S6 and Supplementary Table S3).

**FIGURE 9:**
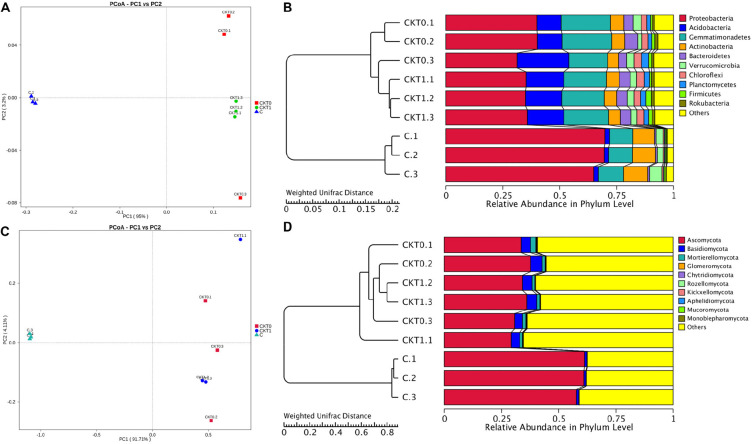
Principal coordinate analysis (PCoA) and unweighted pair-group method with arithmetic mean (UPGMA) dendrogram of bacterial and fungal communities based on weighted unifrac distance. Soils amended with and without catalpol were incubated for 60 days at 26°C at 50% humidity. CKT0 and CKT1 represent control soil treatments without catalpol and collected at experimental initiation (T0) and termination (T1 at 60 days) of incubation, respectively. C represents treated soil amended with catalpol for 60 days and collected for analysis. Results represent the means of three replicates. **(A)** Principal coordinate analysis (PCoA) of bacterial communities in CKT0, CKT1, and C. **(B)** Unweighted pair-group method with arithmetic mean (UPGMA) dendrogram of bacterial communities in CKT0, CKT1, and C. **(C)** Principal coordinate analysis (PCoA) of fungal communities in CKT0, CKT1, and C. **(D)** Unweighted pair-group method with arithmetic mean (UPGMA) dendrogram of fungal communities in CKT0, CKT1, and C.

The bacterial and fungal diversity and richness indices were calculated to reveal within-sample diversity (alpha diversity) (Supplementary Table S4). In contrast to the controls, the soil accumulating catalpol (C) showed significantly lower levels of bacterial and fungal diversity and species richness with observed species, Shannon, Chao1and abundance-based coverage estimator (ACE) indices (*p* < 0.05). Moreover, no significant difference was observed between soil treatments CKT0 and CKT1, indicating the microbial community in control soils was relatively stable during incubation and each control could be further statistically analyzed together with treatment (C) to profile the specific microbes responding to catalpol. The principal coordinate analysis (PCoA) based on weighted UniFrac metric (WUF) was performed to investigate the beta diversity patterns between microbial communities. In the WUF PCoAs, the group of soils accumulated with catalpol was clearly separated from two controls (Figures 9A,C). Unweighted pair-group method with arithmetic mean (UPGMA) clustering based on WUF revealed obvious differences of microbial community between controls and treatment (Figures 9B,D). The bacterial community was comprised mainly of phyla of *Proteobacteria*, *Actinobacteria*, *Gemmatimonadetes*, *Acidobacteria*, *Bacteroidetes*, *Verrucomicrobia*, *Chloroflexi*, *Planctomycetes*, *Firmicutes*, and *Rokubacteria* (Figure 9C), and the fungal community was comprised mainly of phyla of *Ascomycota Basidiomycota Mortierellomycota, Glomeromycota, Chytridiomycota, Rozello mycota, Kickxellomycota, Aphelidiomycota, Mucoromycota, and Monoblepharomycota* (Figure 9D).

**FIGURE 10:**
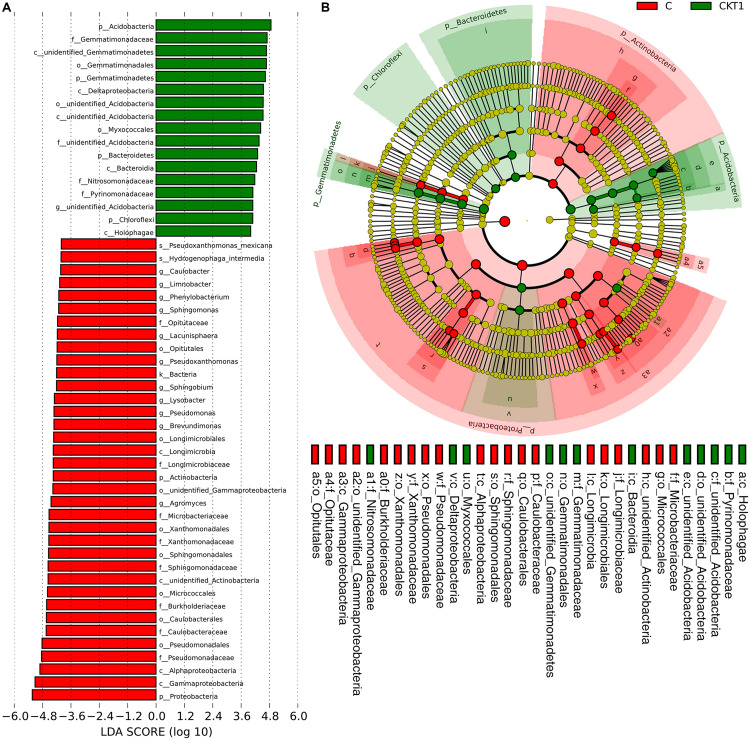
Linear discriminant analysis identified the differentially abundant bacterial taxa between soils amended with and without catalpol. **(A)** Bacterial taxa meeting a linear discriminant analysis (LDA) significant threshold > 4 are presented. **(B)** Cladogram of control and catalpol treatments. CKT1 represents soil control without catalpol. C represents soil amended with catalpol for 60 days.

**FIGURE 11:**
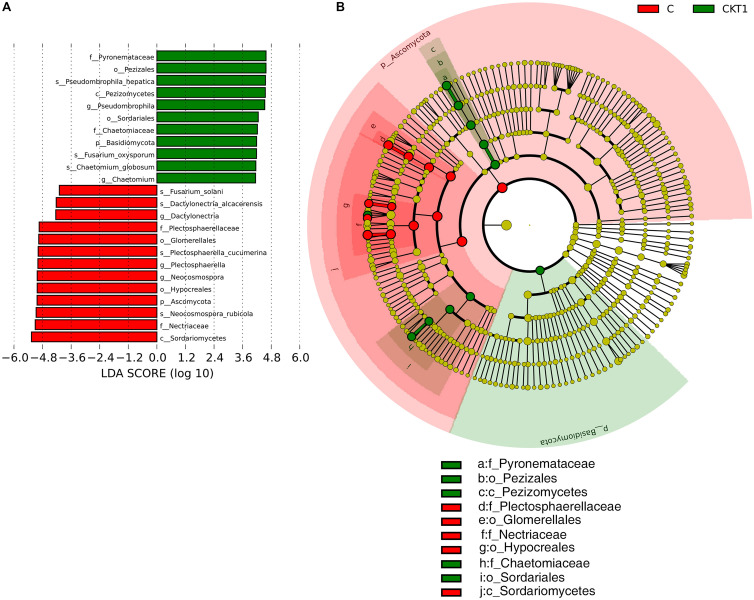
Linear discriminant analysis identified the differentially abundant fungal taxa between soils amended with and without catalpol. **(A)** The fungal taxa meeting a linear discriminant analysis (LDA) significant threshold > 4 are shown. **(B)** Cladogram of control and catalpol treatments. CKT1 represents control soil without catalpol. C represents soil amended with catalpol for 60 days.

The linear discriminant analysis effect size (LEFSe) method was applied to further analyze the differential abundance of soil microbial taxa between controls (CKT0, CKT1) and catalpol treatment (C). Not surprisingly, no significant differences were observed between CKT0 and CKT1 in bacterial nor fungal communities. However, the microbial communities between CKT1 (or CKT0) and C were significantly different. The linear discriminant analysis (LDA) and cladogram showed that the dominant bacterial taxa of CKT1 included the phyla of *Acidobacteria*, *Gemmatimonadetes*, *Bacteroidetes* and *Chloroflexi*, the classes of unidentified *Gemmatimonadetes*, *Deltaproteobacteria*, unidentified *Acidobacteria*, *Bacteroidia* and *Holophagae*, the orders of *Gemmatimonadales*, unidentified *Acidobacteria* and *Myxococcales*, the families of *Gemmatimonadaceae*, unidentified *Acidobacteria*, *Nitrosomonadaceae*, and *Pyrinomonadaceae*, and the genus of unidentified *Acidobacteria*. In contrast, the bacterial phylum of Proteobacteria, the classes of *Gammaproteobacteria*, *Alphaproteobacteria*, unidentified *Actinobacteria* and *Longimicro bia*, the orders of *Pseudomonadales*, *Caulobacterales*, *Micro coccales*, *Sphingomonadales*, *Xanthomonadales*, unidentified *Gammaproteobacteria*, *Longimicrobiales* and *Opitutales*, the families of *Pseudomonadaceae*, *Caulobacterales*, *Burkholderia ceae*, *Sphingomonadaceae*, *Xanthomonadaceae*, *Microbact eriaceae*, *Longimicrobiaceae* and *Opitutaceae*, and the genera of *Agromyces*, *Brevundimonas*, *Pseudomonas*, *Lysobacter*, *Sphing obium*, *Pseudoxanthomonas*, *Lacunisphaera*, *Sphingomonas*, *Phenylobacterium*, *Limnobacter*, and *Caulobacter* were significantly enriched in the soil accumulated with catalpol (Figure 10). For the fungi, the phylum of *Ascomycota*, the class of *Sordariomycetes*, the orders of *Hypocreales* and *Glomerellales*, the families of *Nectriaceae* and *Plectosphaerellaceae*, the genera of *Neocosmospora*, *Plectosphaerella* and *Dactylonectria*, and the species of *Neocosmospora rubicola*, *Plectosphaerella cucumerina*, *Dactylonectria alcacerensis*, and *Fusarium solani* were significantly more abundant in the soil accumulated with catalopl. By comparison, the phylum of *Basidiomycota*, the class of *Pezizomycetes*, the families of *Pyronemataceae* and *Chaetomiaceae*, the orders of *Pezizales* and *Sordariales*, the genera of *Pseudobrophila* and *Chaetomium*, and the species of *Pseudombrophila hepatica*, *Fusarium oxysporum*, and *Chaetomium globosum* showed significantly higher abundance in the soil accumulated without catalpol (Figure 11).

### The Impacts of Accumulated Catalpol on Rhizospheric Microbial Functional Potentials

The phylogenetic investigation of communities by reconstruction of unobserved states (PICRUSt) and the fungal functional guild (FUNGuild) strategies were used to predict their functional shifts resulting from the accumulation of catalpol in *R. glutinosa* rhizosphere, respectively (Langille et al., 2013; Nguyen et al., 2016). Kyoto encyclopedia of genes and genomes orthology (KO) analysis showed that the bacterial genes were mainly assigned to the pathways (at level 2) related to amino acid metabolism, membrane transport, carbohydrate metabolism, replication and repair, energy metabolism, poorly characterized, translation, metabolism of cofactors and vitamins, lipid metabolism and cellular processes and signaling (Supplementary Figure S7A). A visible difference of fungal functional potentials between the controls and treatment (e.g., “Saprotroph” and “Pathotroph”) (Supplementary Figure S7B) was shown in barplot based on FUNGuild analysis. The principal components analysis also clearly showed distinct differences in functional potentials of bacterial and fungal communities between the soils accumulated with (C) and without catalpol (CKT0 and CKT1) (Supplementary Figure S8).

**FIGURE 12:**
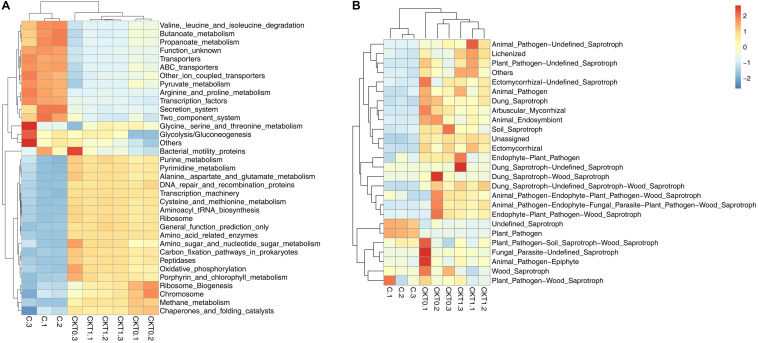
Predicted bacterial **(A)** and fungal **(B)** functional analysis of soil amended with or without catalpol. Soil treatments were incubated for 60 days at 26°C at 50% humidity. CKT0 and CKT1 represent control soil without catalpol and collected at experimental initiation (T0) and termination (T1), respectively. C represents soil amended with catalpol for 60 days. Experimental means are the average of three replicates.

**FIGURE 13:**
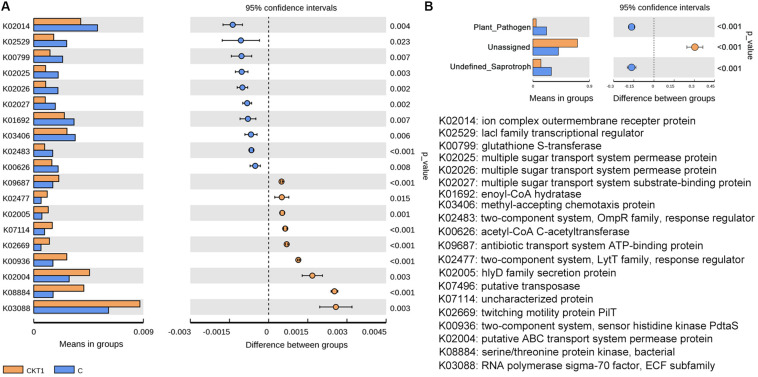
Barplots for significantly different KO categories based on bacterial **(A)** and fungal **(B)** sequences. CKT1 and C represent soil amended catalpol, respectively.

Further microbial functional potentials were profiled and the top 35 bacterial KO pathways assigned at hierarchy level 3 and all fungal functions assigned under trophic mode were analyzed and visualized in heatmaps (Figure 12). For the predicated bacterial potential functions, comparison with CKT0 and CKT1, seven pathways on cellular processes (e.g., “Transporters,” “Pyruvate metabolism,” “Secretion system,”) and nine pathways on environmental information processing (e.g., “Valine leucine and isoleucine degradation,” “Other ion coupled transporters,” “Butanoate metabolism”) were more abundant in the soil accumulated with catalpol. In contrast, nine other pathways on cellular processes (e.g., “Oxidative phosphorylation,” “Ribosome,” “Peptidases”), nine pathways on environmental information processing (e.g., “Transcription machinery,” “Aminoacyl tRNA biosynthesis,” “Carbon fixation pathways in prokaryotes”), and one pathway on “Porphyrin and chlorophyll metabolism” were less abundant. Further statistical analysis using *T*-test more clearly showed the top 10 frequently represented KO categories in the soil accumulated with catalpol. They were engaged in iron complex outermembrane transportation (K02014), regulation of laci family transcription (K02529), xenobiotics biodegradation and metabolism (K00799), multiple sugar transport system (K02025, K02026, K02027), two-component system (K03406, K02483), as well as CoA biosynthesis (K00626, K01692). Ten other 10 categories were significantly less represented in comparison with the control. These were associated with DNA binding (K03088), protein kinases (K08884, K00936), ABC transport system permease protein (K02004), genetic information processing (K07496), hlyD family secretion protein (K02005), LytT family response regulator (K02477), antibiotic transport system ATP-binding protein (K09687) and other biological process (K02699, K07114) (Figure 13A). For the predicated fungal potential functions, “Plant pathogen” and “undefined saprotroph” appeared to be more abundant in the soil accumulated with catalpol (Figure 13B).

### Isolation of *R. glutinosa* Root Specific Microbes and Their Potential Interactions With Catalpol

**FIGURE 14:**
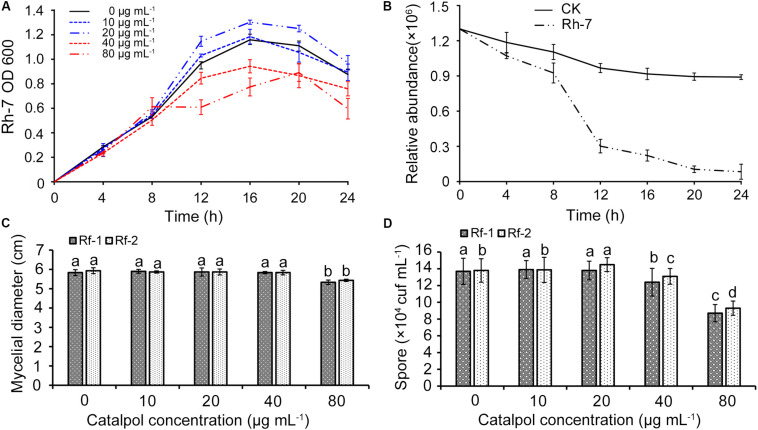
Effects of key *R. glutinosa* root exudate (catalpol) on growth of strain Rh7, Rf1, and Rf2. **(A)** Growth curves of strain Rh7 under different dosages of catalpol. **(B)** Quantification of catalpol in the cultures (20 μg mL^–1^) incubated with and without Rh7. **(C)** Mycelial diameter of strain Rf1 and Rf2 in the plates containing different dosages of catalpol. **(D)** Spores of strain Rf1 and Rf2 in the plates containing different dosages of catalpol. Different letters indicate statistical difference (*p* < 0.05).

A total of nine bacterial (named Rh1-Rh9) and three fungal strains (named Rf1-Rf3) were successfully isolated from newly planted and replanted *R. glutinosa* rhizosphere soils, respectively. 16S rRNA and ITS region sequencing revealed that bacterial strain Rh7, fungal strain Rf1 and Rf2 belonged to *Pseudomonas aeruginosa*, *Fusarium oxysporum* and *Fusarium solani*. These three strains were selected for further investigation since they were classified to the genera which were specifically profiled in the soil known to accumulate key *R. glutinosa* root metabolites through exudation (catalpol). Moreover, the genera *Pseudomonas* and *Fusarium* have been reported as the taxa significantly shifting in rhizosphere soils of *R. glutinosa* under various replantation regimes (Wu et al., 2018a,b,c). Pathogenic assay of these strains on *R. glutinosa* tissue culture seedlings showed that Rf1 (*F. oxysporum*) and Rf2 (*F. solani*) efficiently caused wilt disease in seedlings and a noticeable symptom was observed by 6 days following inoculation. In contrast, strain Rh7 (*P. aeruginosa*) showed no pathogenic activity on the seedlings, but delayed the onset of pathogenic strains Rf1 and Rf2 to *R. glutinosa* (Supplementary Figure S3). A clear inhibition zone of Rh7 against Rf1 and Rf2 hyphal growth also suggested its potential for use as a biocontrol (Supplementary Figure S2). Cultures of strain Rh7 (control culture and cultures exposed to different dosages of catalpol) were incubated and monitored spectrophotometrically. For each catalpol concentration, similar OD_600_ patterns were observed during the first 8 h. Cultures with dosages of 10 and 20 μg mL^–1^ catalpol showed an increase in the OD_600_ during 8–12 h, while declining trends OD_600_ were observed in the cultures at dosages of 40 and 80 μg mL^–1^ catalpol (Figure 14A), suggestive of a potential hormesis-like effect of catalpol on Rh7. Comparison with control cultures (20 μg mL^–1^ catalpol) and those with inoculation of Rh7, the relative abundance of catalpol in the cultures inoculated with Rh7 rapidly decreased at 8–12 h and remained stable after 20 h (Figure 14B), suggesting that Rh7 is likely involved in the utilization, biodegradation and/or transformation of catalpol. When observing hyphal growth of strain Rf1 and Rf2 on the plates containing different concentrations of catalpol, neither significant inhibition or promotion were shown in 40 μg mL^–1^, but a significant inhibition in growth was observed at concentrations over 80 μg mL^–1^. Moreover, sporulation in strain Rf1 and Rf2 in cultures at 40 and 80 μg mL^–1^ catalpol was significantly decreased (Figure 14C). Greater numbers of Rf2 spores were detected in cultures containing 20 μg mL^–1^ than those cultivated with 0 and 10 μg mL^–1^ catalpol (Figure 14D).

## Discussion

Root exudates typically contain mucilage, proteins, amino acids, organic acids, sugars, phenolics and various secondary metabolites that represent a significant carbon cost to the plant in terms of production and exudation (Uren, 2000; Bais et al., 2008). Dynamic interactions between plant-plant and plant-soil microbes are regulated based on the presence or absence of various metabolites (Bais et al., 2006; Kong et al., 2018; Stringlis et al., 2018). Evidence has also accumulated that now clear suggests that bioactive constituents in root exudates play important roles in replant problems associated with perennial plants (Young, 1984; Asao et al., 2003; Hofmann et al., 2012). Our previous studies have primarily investigated the soil physicochemical properties following replanting (Zhang Z. et al., 2010), the restructured microbiome in the plant rhizosphere following replanting (Wu et al., 2015, 2016, 2018a,b; Bao et al., 2016), and physiological and biochemical responses of *R. glutinosa* under consecutive monocultures (Yang et al., 2011; Li et al., 2015; Chen et al., 2018; Wang et al., 2019). However, the quantifiable release of bioactive secondary products by *R. glutinosa* roots through root exudation and decomposition is regarded as a critical step impacting the rhizosphere ecosystem and subsequent growth and development of the plant, but specifics of this process remain unclear and are currently limited by our lack of knowledge about the root exudation process itself in this perennial plant and the specific chemical composition of the exudate. Hence, the reported studies were conceived to further study the living *R. glutinosa* root system.

By selectively employing stereoscopic light microscopy, we observed considerable quantities of yellow-colored exudates accumulating at root hairs, secondary root tips and in the periderm of living roots of 7-day-old *R. glutinosa* seedling (Figure 1A). Very dark yellow or golden colored metabolites were also observed in the periderm of the mature tubers. To minimize the potential impacts of external environmental factors on chemical properties of *R. glutinosa* root released products, root exudates were collected by rapidly dipping *R. glutinosa* root hairs into methanol (Zhu et al., 2016). Simultaneously, the rhizosphere soil of *R. glutinosa* from production sites within its geo-authentic region were contrasted with control soil from nearby field unplanted with *R. glutinosa* and both were immediately extracted in methanol after collecting. By employing sensitive cutting edge metabolic profiling strategies, we were able to identify for the first time several unique metabolites that accumulated in significant quantities in the *R. glutinosa* rhizosphere (Zhang et al., 2019).

A personal compound datebase library was designed and utilized for accurate identification of *R. glutinosa* root exudates based on the retention time, accurate mass, mass spectra and other molecular features of these metabolites. In this study, non-targeted metabolic profiling with LC-MS QToF instrumentation and Venn three-factor visualization clearly showed that eleven compounds were detected in both root exudates and rhizosphere soil but were absent in uninfested control soil, suggesting these metabolites were sourced from *R. glutinosa* and accumulated over time in the surrounding rhizosphere. By searching the molecular features of metabolites of interest against our PCDL and comparing this to known analytical standards, one iridoid glycoside (catalpol) and six phenylethanoid glycosides (acteoside, isoacteoside, leucosceptoside A, 2′-actetylacetoside, martynoside, 2,4“Di-O-acetyl-3” ′-verbascoside) were positively identified. Subsequent quantification analysis of these identified metabolites in crude *R. glutinosa* root exudates and extracts of rhizosphere soils showed that catalpol and acteoside were two most abundant root-released metabolites. Increasing levels of accumulation of these compounds were observed in the rhizosphere over time with crop maturity. The concentration of catalpol and acteoside peaked at 12.6 and 7.87 μg g^–1^ at the middle-stage of tuber expansion or at crop maturity (the harvest stage), respectively. Additionally, catalpol abundance was significantly higher than that of acteoside both in *R. glutinosa* root exudates and associated rhizosphere soils.

Catalpol and acteoside are known to be the most important biomedicinally active constituents in *R. glutinosa* and are typically used as quality control markers for related tuber products by the Chinese Pharmacopoeia (Commission, 2017). These metabolites have also previously been actively investigated in pharmacology (Shieh et al., 2011; Wang et al., 2016). The distribution of catalpol and acteoside in radial (mainly including xylem) and non-radial striation (including phloem and periderm) of *R. glutinosa* tuber was preliminarily investigated in 2018 for the purpose of establishing supplementary markers for quality control of the corresponding products (Zhi et al., 2018). However, the present study has provided detailed information regarding catalpol and acteoside accumulation and secretion by living plants and assessed their activity as allelochemicals or phytotoxins. In addition, we analyzed their concentration in xylem, phloem, periderm and secondary roots of *R. glutinosa* tubers.

Results showed catalpol and acteoside presented in all below-ground tissues, and highest levels were detected in the periderm and secondary roots. Past studies on *Brassica napus* (Mccully et al., 2008) and *Echium plantagineum* (Zhu et al., 2016) have also demonstrated that periderm and root hairs are the important sites of release of phytotoxins including the allelochemical families of shikonins and glucosinolates. Thus, we postulated and demonstrated by numerous assays that the direct release of the iriod and phenylethanoid glycosides occurred in both the periderm and by living root hairs of *R. glutinosa* through exudation. Recent evidence has also suggested that the metabolites released by root exudates of plants including *Cunninghamia lanceolate*, *Asparagus officinalis* and *Glycyrrhiza uralensis* are implicated in replant failure associated with replanting (Kong et al., 2008; Yeasmin et al., 2014; Xia et al., 2017). In this study, catalpol and acteoside exhibited significant phytotoxicity in two sensitive bioindicator species, *Lepidium sativum* L. (dicotyledon) and *Lolium perenne* L. (monocotyledon) in two different bioassays. Both metabolites also negatively impacted radical growth and development of *R. glutinosa* seedlings in our recent study (Zhang et al., 2019), further supporting the hypothesis that the replant problem of *R. glutinosa* may also be associated with the autotoxicity caused by catalpol and acteoside. In contrast to the IC_50_ of catalpol on radicle growth of several dicots (0.82 mg mL^–1^ for *R. glutinosa* seeds and 0.81 for *L. sativum* seeds), the monocot (e.g., IC_50_ of catalpol on *L. perenne* seeds was 1.34 mg mL^–1^) was less sensitive to catalpol. Interestingly, the monocots (e.g., *Zea mays* and *Triticum* spp.) were also specifically suggested as less sensitive rotational crops in *R. glutinosa* production systems (Zhang et al., 2019).

Notably, previous quantification of catalpol and acteoside in fresh tubers (85.2% water content) of *R. glutinosa* at harvest stage was as high as 33.33 and 0.28 mg g^–1^, respectively (Wang et al., 2015). Further field investigation found considerable quantities of tuber residues (125.9 g m^–2^, dry weight) were retained in cultivated land after harvesting (Zhang et al., 2019). Therefore, we suggest that the growth of *R. glutinosa* was reduced in monoculture conditions at least in part due to the continual accumulation of catalpol and other related phenylethanoid glycosides originating from active root exudation and residue decomposition over time. Strikingly, modified Parker assay showed that the phytotoxicity of catalpol and acteoside was greater in pasteurized soil than that in non-pasteurized soil, and the half-life of catalpol and acteoside in pasteurized soil was also significantly longer in contrast to the control. Reduction in phytotoxicity is usually achieved when soil microbes degrade/transform allelochemicals into less toxic forms (Ghulam et al., 2008; Kobayashi, 2010). Thus, specific microbes potentially recruited by the presence of *R. glutinosa* root exudates may dramatically impact the phytotoxicity of catalpol due to microbial utilization, biodegradation and/or transformation (Inderjit., 2005; Cipollini et al., 2012; Mishra et al., 2013).

In this study, six phenylethanoid glycosides (acteoside, isoacteoside, leucosceptoside A, 2′-actetylacetoside, martynoside, 2,4“Di-O-acetyl-3” ′-verbascoside) were detected in the root exudates and rhizosphere soil of *R. glutinosa*, and were present in high abundance in contrast to previous reports suggesting the prevalence of phenolic acids in the rhizsosphere. The core structures of phenylethanoid glycosides are often substituted with such common constituents as caffeic acid, coumaric acid, cinnamic acid, ferulic acid, and isoferulic acid. Importantly, most phenylethanoid glycosides are unstable at alkaline and acidic conditions (Zhou et al., 2017). Thus, we deduced that the phenolic acids previously detected in the *R. glutinosa* infested soils likely originated from the presence of phenylethanoid glycosides such as those observed in this study. Due to the significantly stronger phytotoxic activity and higher abundance of catalpol in *R. glutinosa* root exudates and rhizosphere soils, we conducted additional studies on the bioactivity of catalpol in this investigation, noting that further investigations of biodegradation and rhizo-ecological processes of phenylethanoid glycosides will be also performed in the future.

Microbial profiling of the soil amended with and without catalpol showed that several microbial species or genera responded to the accumulation of catalpol in soil over time. Specifically, *Chaetomium globosum*, *Fusarium oxysporum*, and *Pseudombrophila hepatica* were significantly suppressed in amended soils in contrast to the control. In past studies, *F. solani*, *P. cucumerina*, *N. rubicola*, and *D. alcacerensis* were identified as phytopathogens causing leaf spot, root and stem rot (Garibaldi et al., 2013; Huo et al., 2018; Langenhoven et al., 2018; Zheng et al., 2018), and some enriched bacterial genera including *Agromyces*, *Pseudoxanthomonas* (e.g., *P. mexicana*), *Phenylobacterium*, *Caulobacter*, and *Sphingomonas* were also widely reported to participate in biodegradation of aromatic hydrocarbons such as di-(2-ethylhexyl) phthalate, benzo(a)anthracene, chrysene, o-terphenyl and phenanthrene (Leys et al., 2004; Tao et al., 2007; Lladó et al., 2009; Zhao et al., 2016; Lu et al., 2019), suggesting these genera probably accounted for the upregulated microbial functions of plant pathogen (Figure 13B) and xenobiotic biodegradation and metabolism (Figures 12A, 13A), respectively.

Additionally, the *Lysobacter* is regarded as a genus of producing rich source of antibiotics for associated with defense in plant pathogens including *Aspergillus niger*, *Rhizoctonia solani*, *Phytophthora capsici*, and *Fusarium oxysporum* (Xie et al., 2012; Yong et al., 2013; Ruth et al., 2015). However, at this stage, our understanding of the functional potential of a given genera is still limited due to incomplete identification of all associated species, and our inability to successfully culture many soil microbes. For examples, only a few species have been identified as thiosulfate-oxidizing, heterotrophic bacterium in the genera of *Limnobacter* (Spring et al., 2001; Lu et al., 2011; Chen et al., 2016); the members of *Pseudomonas* demonstrate considerable metabolic diversity and consequently are able to occupy a wide range of niches (Madigan et al., 1997), including the plant pathogen *Pseudomonas syringae* and the plant growth-promoting *Pseudomonas fluorescens* (Buell et al., 2003; Paulsen et al., 2005). To further profile the potential roles of shifted microbial communities in regulating *R. glutinosa*-soil feedbacks, the previous literature was first reviewed and several of the root specific microbes previously associated with *R. glutinosa* were isolated for further assays. We determined by literature review that a species in genera of *Brevundimonas* (*Brevundimonas diminuta*) showed growth promoting effects on *Oryza sativa* (Singh et al., 2016); *Sphingomonas* S3-4 in genera of *Sphingomonas* had the ability to completely eliminate the production of mycotoxin deoxynivalenol of phytopathogen *Fusarium* (He et al., 2017). Interestingly, *Sphingobium* sp. strain JS1018, which was isolated from rhizosphere of *Arachis hypogaea* L., showed a biodegradation effect on a stilbene allelochemical-pterostilbene released from *A. hypogaea* important in resistance of fungal pathogens (Yu et al., 2019). Thus, we believed that the members of genera of *Pseudomonas*, *Agromyces*, *Pseudoxanthomonas*, *Phenylobacterium*, *Caulobacter*, *Lysobacter*, *Brevundimonas*, *Sphingobium*, and *Sphingomonas* were colonized in soil accumulating significant levels of catalpol potentially because of their active chemotaxis and/or their successful interspecific competition indirectly mediated by catalpol.

In addition, some species in these genera are likely involved in the direct biodegradation of catalpol. Based on this inference, we then isolated the root specific microbes in both newly planted and replanted *R. glutinosa*. A bacterial strain Rh7 was specifically screened in the rhizosphere of newly planted *R. glutinosa* and identified as *Pseudomonas aeruginosa*. Notably, significant *in vitro* promotion of strain Rh7 (OD_600_ value) using the dosages of 10 and 20 μg mL^–1^ catalpol was observed during its logarithmic phase (8–12 h). Additionally, an inhibitory effect of catalpol on strain Rh7 growth was found at the concentrations of 40 and 80 μg mL^–1^. Further quantification of catalpol in treated aliquots showed rapid degradation of catalpol between 8 and 12 h, suggesting strain Rh7 was directly involved in the utilization, biodegradation and/or transformation of catalpol at the concentration of 20 μg mL^–1^. The inhibition of strain Rh7 growth at 40 and 80 μg mL^–1^ of catalpol might be explained due to its presence at high concentrations. Inoculation of *Sphingobacterium* sp. PG-1 in soil in monoculture with *Panax ginseng* alleviated *P. ginseng* replant problem by degrading phytotoxic diisobutyl phthalate by 79.9% and reducing ginseng death by 40.1%, respectively (Dong et al., 2018b), suggests that further utilization of catalpol-degrading bacteria (e.g., strain Rh7) is one method to potentially alleviate the autotoxicity of *R. glutinosa*.

Furthermore, two fungal strains Rf1 and Rf2 were isolated from rhizosphere soil of replanted *R. glutinosa* (RP2) and identified as *Fusarium oxysporum* and *Fusarium solani*, respectively. Significant pathogenicity of Rf1 and Rf2 on *R. glutinosa* tissue culture seedlings was observed, and *F. solani* was reported as the potential pathogen causing root decay of *R. glutinosa* (Wang et al., 2018). *Fusarium oxysporum* was also identified as a host specific pathogen of *R. glutinosa* (Li et al., 2013). By counting spore numbers of the strain Rf1 and Rf2 in the cultures containing 0–80 μg mL^–1^ of catalpol, we found catalpol promoted spore proliferation of Rf2 only at 20 μg mL^–1^. Moreover, significant inhibition of catalpol on hyphal growth (colony diameter value) and spore proliferation were observed at the dosage of 80 μg mL^–1^. Notably, co-cultivation of isolated bacterial strain Rh7 (*Pseudomonas aeruginosa*) showed no pathogenic activity on the *R. glutinosa* seedlings, but significantly delayed the morbidity of seedlings infected by strains Rf1 (*F. oxysporum*) and Rf2 (*F. solani*).

In profiling of the soil with accumulated catalpol, relative lower abundance of *F. oxysporum* was observed and this could have resulted from the antagonism effects of members of genus of *Pseudomonas* (e.g., *P. aeruginosa*) whose numbers were promoted in this treatment. Greater abundance of *F. solani* could be associated with the growth promotion effects of catalpol and relative lower biocontrol effects of *P. aeruginosa* against *F. solani* (Supplementary Figure S3). Nevertheless, field investigation of growth status of *R. glutinosa* under different monocultures showed that the survival rate decreased form 96.6% in newly planted field to 27.3% in the soil obtained from 2 years of replanting and corresponding root dry weight per m^2^ dramatically decreased as well. Catalpol concentration in the rhizosphere soils of *R. glutinosa* also significantly increased from 11.40 μg g^–1^ (in newly planted field) to 23.10 μg g^–1^ (in soil replanted for 2 years) (Supplementary Figure S9). Commonly, the sign of positive and negative feedbacks is determined by the relationship between the microbe’s competitive ability in the rhizosphere and the effect of that microbe on that plant (Bever et al., 2012). In the previous studies, we have found the genera of *Pseudomonas* and *Lysobacter* were significantly decreased, while the relative abundance of *Fusarium* was remarkably enriched in the replanted soil than that in the newly planted soil of *R. glutinosa* (Wu et al., 2018a,b,c). The large amount of *R. glutinosa* leaf and tuber residues present in the field after harvest likely resulted in rapid enrichment of catalpol in the soil rhizosphere (Zhang et al., 2019). Thus, the reduced levels of *Pseudomonas* and enriched *Fusarium* in replanted *R. glutinosa* rhizospheres could be potentially associated with effects of high catalpol concentration on *Pseudomonas* and in response the biocontrol effects against pathogens were suppressed.

**FIGURE 15:**
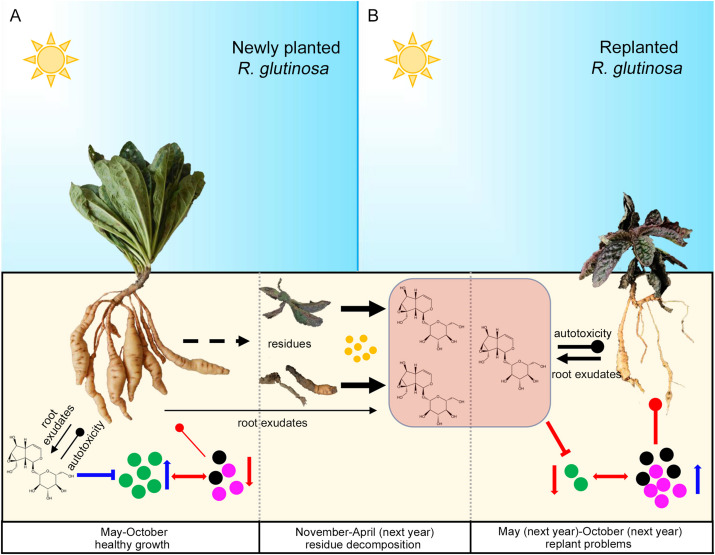
Potential plant-soil feedbacks of *R. glutinosa* under newly planted **(A)** and replanted **(B)** conditions. Newly planted *R. glutinosa* were commonly cultivated from May to October, and harvested in November. Plant residues were abandoned in the field after harvest, and catalpol were released and accumulated in the soil with the decomposition of residues by soil microbes (yellow color) from November to April of next year. *R. glutinosa* was replanted in the same field in May and abnormal growth were observed. Green microbes represented the biocontrol agents or plant growth promoting microbes. Black and purple microbes represented the pathogens. Number of the microbes represented their abundance. Suppression and promotion of microbial growth were represented by red and blue arrow, respectively. Autotoxicity was represented by the thickness of black clubs. Pathogenicity was represented by the thickness of red clubs. The promotion and inhibition effects of catalpol on microbe growth were represented by blue and red “T” lines, respectively.

In summary, in the newly planted field, relatively lower concentrations of metabolites such as catalpol accumulated in the *R. glutinosa* rhizosphere due to the biodegradation effects of root specific microbes (e.g., *P. aeruginosa*), a positive *R. glutinosa*-soil feedback system potentially in play via the preponderant colonization of plant growth promoting strains (e.g., *P. aeruginosa*) and their antibiotic effects on host-specific pathogens (e.g., *F. oxysporum* and *F. solani*). However, catalpol and other bioactive compounds could rapidly increase after harvest due to the *R. glutinosa* residues. Once *R. glutinosa* was propagated in monocultural conditions in the same field, the increased catalpol concentrations may have impact the growth of replanted *R. glutinosa* through its autotoxicity. Simultaneously, the plant growth-promoting rhizobacteria (e.g., *P. aeruginosa*) may be suppressed by high catalpol concentration, in turn, host-specific pathogens dominated the rhizospheric microbial community and potentially were associated with replanting disease (Figure 15).

## Conclusion

We carefully monitored the production and release of *R. glutinosa* root exudates both visually by stereomicroscope and chemically using state of the art metabolic profiling methods, and various seedling bioassays evaluated the phytotoxicity of key metabolites. Results confirmed that the most abundant and phytotoxic metabolite-catalpol was secreted from the tuber periderm, secondary roots and root hairs at various growth stages of *R. glutinosa*. Interactions between *R. glutinosa* root exudates and rhizobiome were novelly investigated by microbial profiling of the soil with accumulated catalpol and by co-cultivation of isolated root specific strains (biocontrol strain Rh7 and pathogenic strains Rf1 and Rf2) with key root exudate (catalpol). Results indicated that, once present in the rhizosphere in abundance, catalpol contributed to and shaped soil microbial composition, and in turn the allelopathic efficacy of catalpol was reversibly impacted depending on the preponderantly colonized rhizobiome regulated by catalpol concentration. Summarily, the key metabolites in *R. glutinosa* root exudates were identified and the rhizobiomes that are likely involved in the root exudate biodegradation and the potential communities that dominate the generation of different plant-soil feedbacks were evaluated. Our findings highlighted the need for accurate identification of plant root exudates to investigate the plant-microbial interactions in the soil rhizosphere. It is critical to understand the underlying mechanisms related to perennial replant problems in numerous traditional Chinese medicines.

## Data Availability Statement

The sequencing data has been uploaded to NCBI-Sequence Read Archive with the submission Number SUB7502869 and BioProject Number PRJNA635384.

## Author Contributions

BZ, LW, ML, LG, and ZZ conceived and designed the experiments. BZ, XZ, BYZ, and LZ performed the experiments. BZ, PW, and LG performed the bioinformatic analysis. BZ and LW wrote the manuscript. All authors contributed to the article and approved the submitted version.

## Conflict of Interest

The authors declare that the research was conducted in the absence of any commercial or financial relationships that could be construed as a potential conflict of interest.
