# Supplementary material for: Rehmannia glutinosa Replant Issues: Root Exudate-Rhizobiome Interactions Clearly Influence Replant Success
Source: Front Microbiol. 2020 Jun 30;11:1413. doi: 10.3389/fmicb.2020.01413 (PMC7344158; doi:10.3389/fmicb.2020.01413)
Supplement: Supplementary file 1 [file Data_Sheet_1.docx]

Supplementary Material

**Supplementary Table S1** Standard curve calibration and linear ranges of the *R. glutinosa* metabolites catalpol and acteoside.

| **Compounds** | **Calibration curve** | ***r*** | **Linear range (μg)** |
| --- | --- | --- | --- |
| Catalpol | y=9.27×10^6^x-4.99×10^3^ | 0.98 | 1.216-18.657 |
| Acteoside | y=6.94×10^6^x-2.74×10^3^ | 0.99 | 0.633-9.732 |


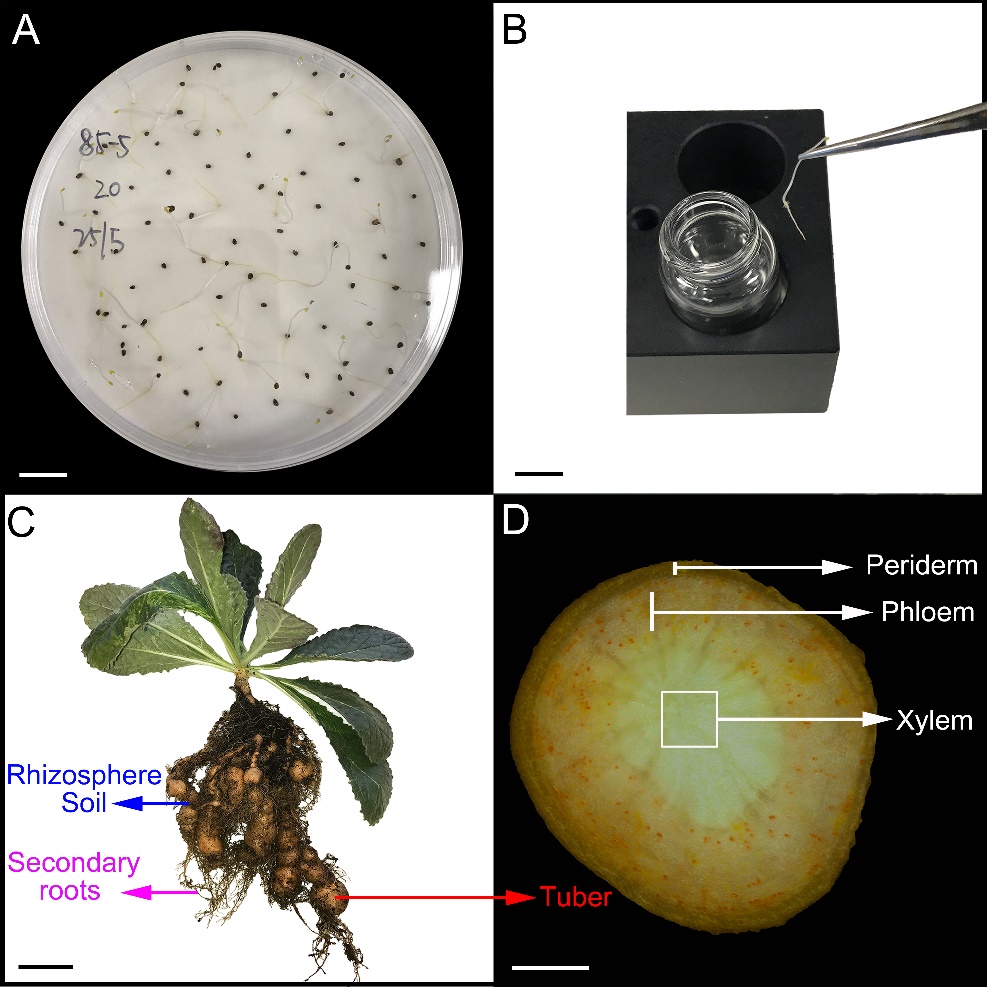


**Supplementary Figure S1.** Images of *R. glutinosa* root exudates and various tuber tissues. A: *R. glutinosa* seedlings at 7 d of age in Petri dish. Bar, 1 cm; B: Collection of root exudates from *R. glutinosa* seedlings by rapid dip method. Bar, 1 cm. C: 120-day-old *R. glutinosa*, showing rhizosphere soil, tubers and secondary roots. Bar, 5 cm. D: Bright field image of tuber cross section of 120-day-old *R. glutinosa*, showing xylem, phloem, and cortical tissues. Bar, 1 cm.

**Supplementary Table S2** Eleven highly abundant metabolites identified in the extracts of *R. glutinosa* rhizosphere soil.

| **Compounds** | **RT (min)** | **m/z calcd** | **Name** | **Formula** | **MS/MS fragment ions** |
| --- | --- | --- | --- | --- | --- |
| 1 | 1.132 | 362.1077 | Catalpol | C_15_H_22_O_10_ | 183.0690 |
| 2 | 6.926 | 390.1859 | Unknown | C_17_H_26_O_10_ | 151.0740 |
| 3 | 7.929 | 390.2595 | Rehmaionoside A/B | C_19_H_34_O_8_ | 211.1694 |
| 4 | 7.95 | 388.1932 | Rehmaionoside C | C_19_H_32_O_8_ | 209.1534 |
| 5 | 8.033 | 624.2401 | Acteoside | C_29_H_36_O_15_ | 471.1502, 325.0925, 163.0389 |
| 6 | 8.239 | 624.2399 | Isoacteoside | C_29_H_36_O_15_ | 471.1643, 325.0856, 163.0451 |
| 7 | 8.572 | 638.2545 | Leucosceptoside A | C_30_H_38_O_15_ | 485.1665, 339.1061, 177.0542 |
| 8 | 8.741 | 666.2510 | 2’-actetylacetoside | C_31_H_38_O_16_ | 513.1601, 367.1036, 163.0391 |
| 9 | 9.267 | 652.2712 | Martynoside | C_31_H_40_O_15_ | 485.1666, 339.1078, 177.0546 |
| 10 | 10.034 | 722.2763 | 2,4“Di-O-acetyl-3” ’-verbascoside | C_34_H_42_O_17_ | 569.1876, 381.1171, 177.0547 |
| 11 | 10.587 | 622.2975 | Unknown | C_29_H_34_O_15_ | 395.0981 |


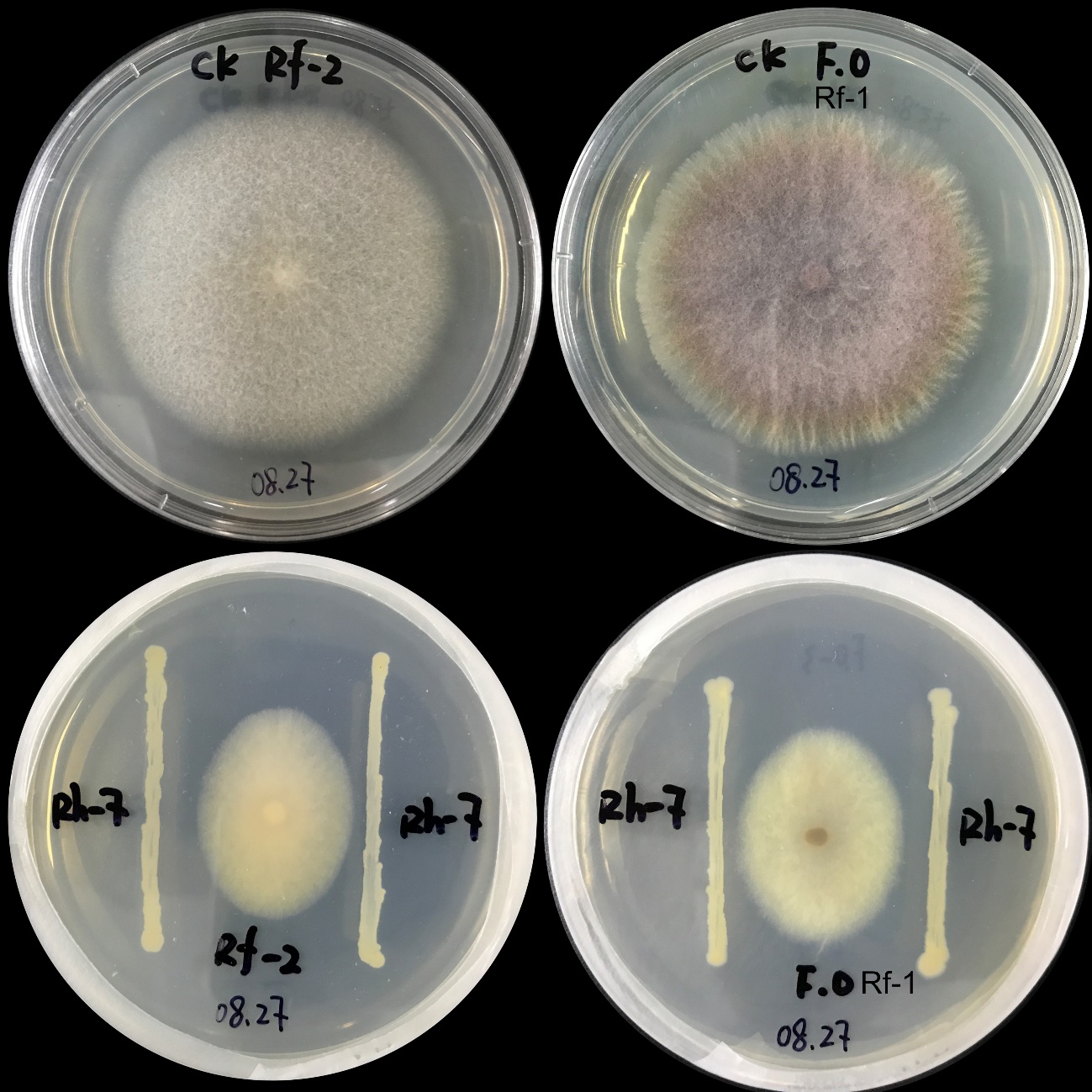


**Supplementary Figure S2.** Assessment of antagonistic activity of isolated strain Rh-7 against strain Rf-1 and Rf-2. Strain Rf-1 and Rf-2 were identified as *Fusarium oxysporum* and *Fusarium solani* by ITS region sequencing, respectively. Strain Rh-7 was identified as *Pseudomonas aeruginosa* by 16S rRNA sequencing.


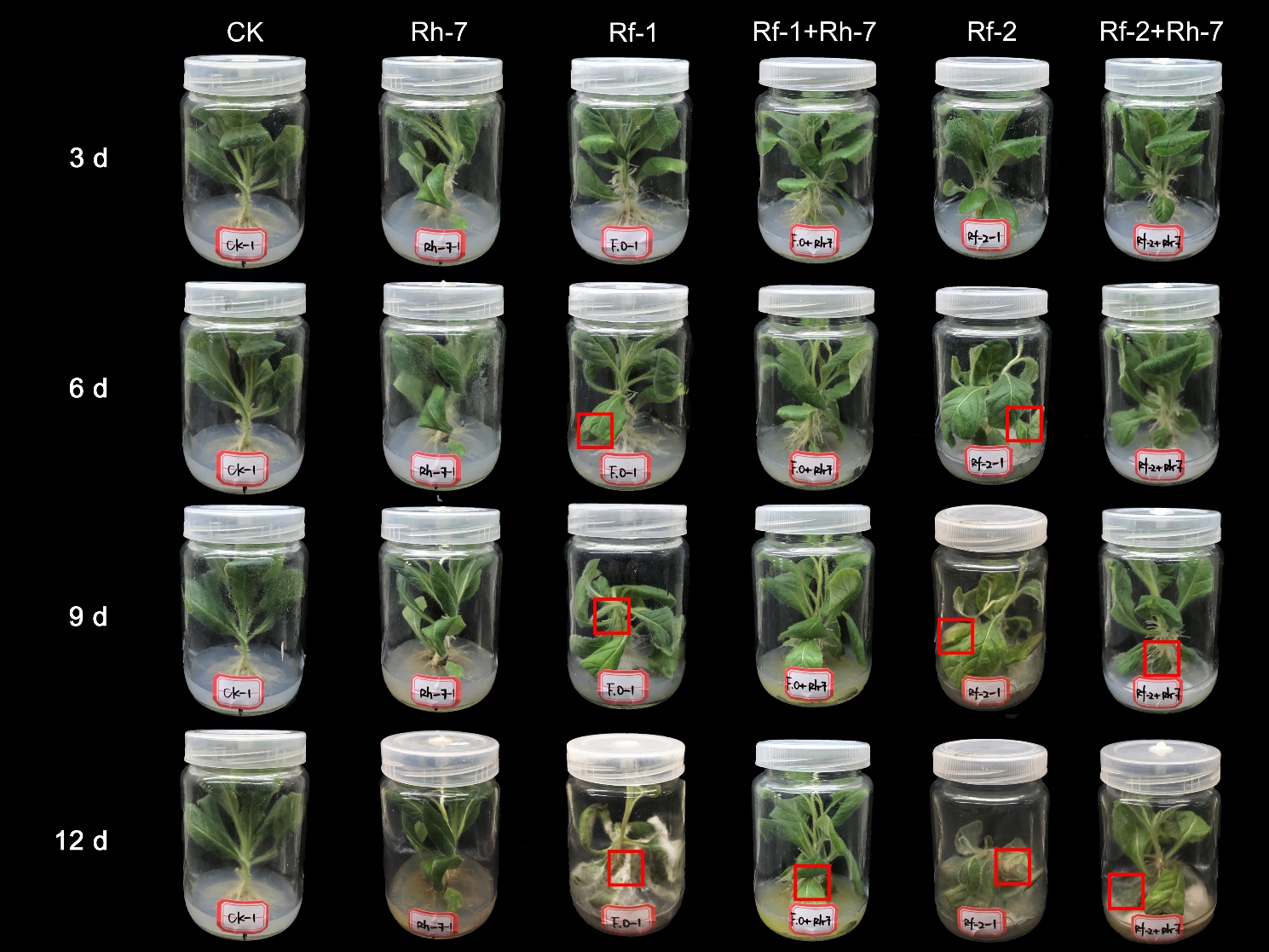


**Supplementary Figure S3.** Assessment of the pathogenicity of isolated strain Rf-1 and Rf-2 and biocontrol potential of strain Rh-7. Strain Rf-1 and Rf-2 were identified as *Fusarium oxysporum* and *Fusarium solani* by ITS region sequencing, respectively. Strain Rh-7 was identified as *Pseudomonas aeruginosa* by 16S rRNA sequencing.


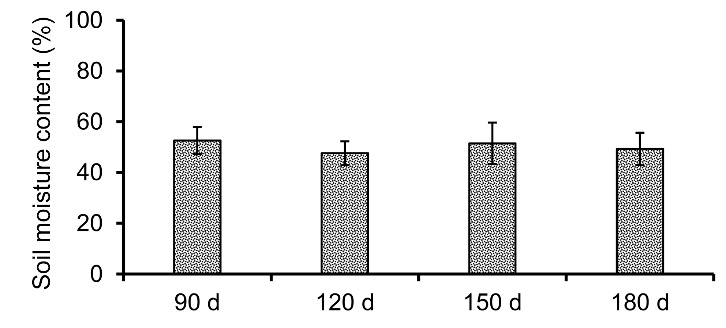


**Supplementary Figure S4.** Moisture content of rhizosphere soils at various *R. glutinosa* growth stages. Four *R. glutinosa* growth stages were screened for moisture content including the initiation of tuber formation, the early stage of tuber expansion, mid stage tuber expansion and tuber formation at harvest, at 90, 120, 150 and 180 d after seed germination, respectively.


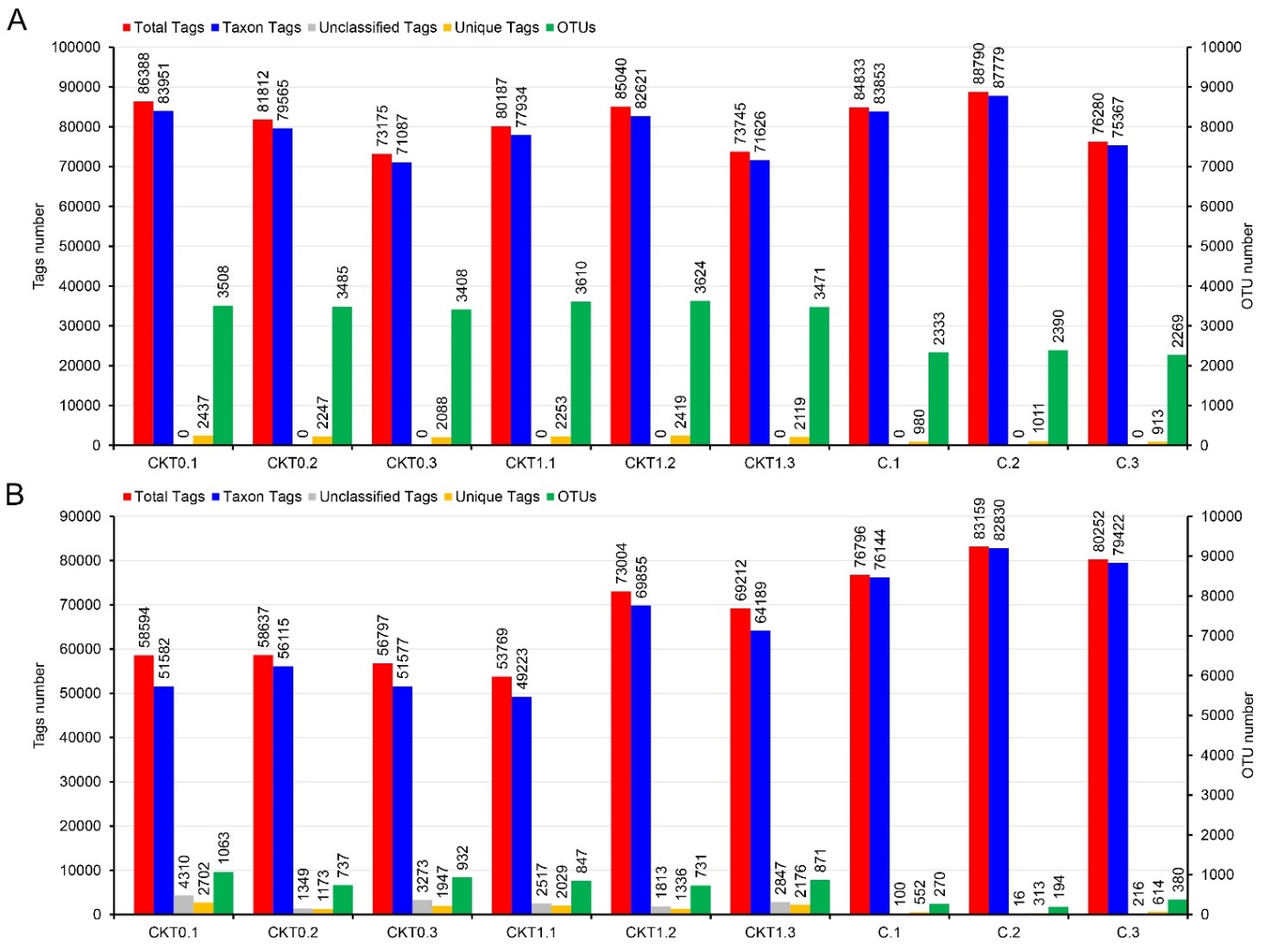


**Supplementary Figure S5.** Cluster analysis of bacterial (A) and fungal (B) OTUs and associated species annotation. Soils amended with or without catalpol were incubated for 60 days at 26 ℃ at 50 % humidity. CKT0 represents control soil without catalpol collected at experimental initiation and CKT1 soil collected at experimental termination, respectively. C represents soil treated with catalpol for 60 days. Means presented are the average of three replicates.


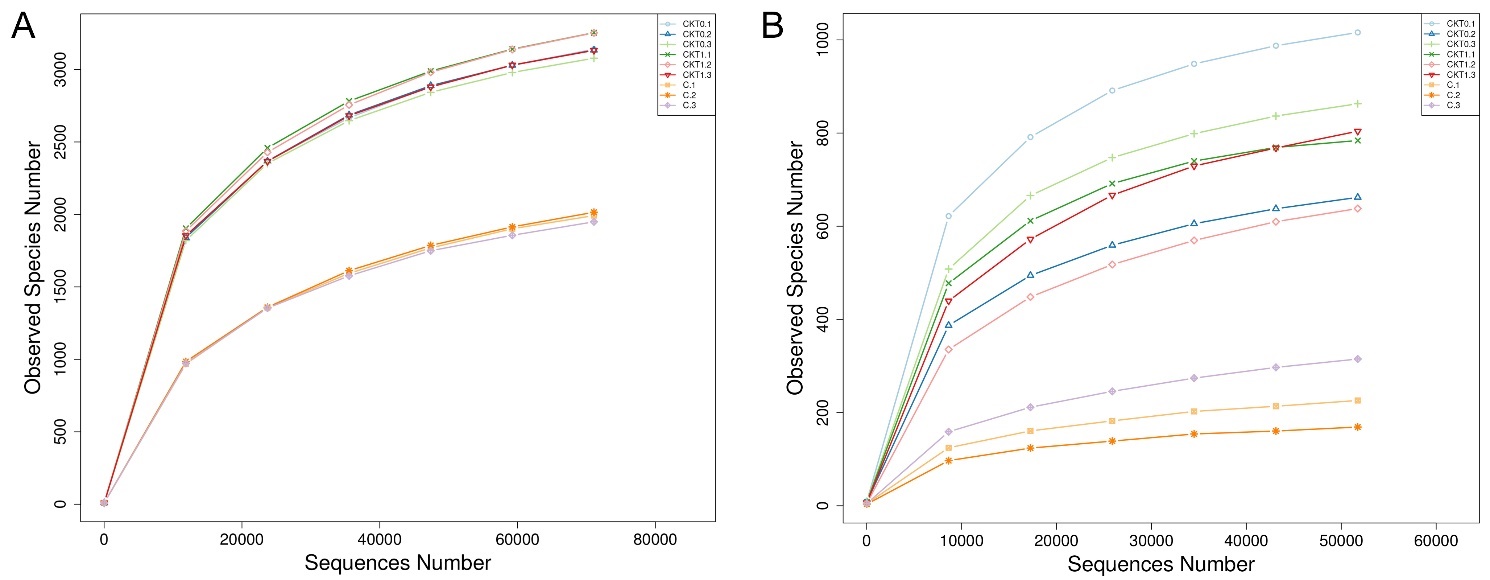


**Supplementary Figure S6.** Rarefaction curves of bacterial and fungal communities based on observed operational taxonomic units (OTUs) at the 97% similarity cut-off level. Soils amended with and without catalpol were incubated for 60 days at 26 ℃ at 50 % humidity. CKT0 and CKT1 represent control soil without catalpol, and soil collected at experimental termination, respectively. C represents soil treated with catalpol for 60 days. Means are the average of three replicates.

**Supplementary Table S3.** Estimation of sequence quality of bacterial and fungal communities in soil amended with and without catalpol by Goods coverage analysis.

| **Goods coverage** | **CKT0** | **CKT1** | **C** |
| --- | --- | --- | --- |
| **Bacteria** | 0.992±0.001 | 0.992±0.001 | 0.993±0.000 |
| **Fungus** | 0.997±0.001 | 0.998±0.001 | 0.999±0.001 |

Soils amended with and without catalpol were incubated for 60 days at 26 ℃ at 50 % humidity. CKT0 and CKT1 represent control soil without catalpol and collected at experimental initiation and soil collected at experimental termination, respectively. C represents soil treated with catalpol for 60 days. Means are the average of three replicates.

**Supplementary Table S4.** Species richness and diversity indices in soils amended with or without catalpol.

|  | **Groups** | **Observed species** | **Shannon** | **Chao1** | **ACE** |
| --- | --- | --- | --- | --- | --- |
| **Bacteria** | CKT0 | 3117.3±34.2^a^ | 9.5±0.1^a^ | 3416.1±104.1^a^ | 3458.0±83.7^a^ |
|  | CKT1 | 3213.0±70.2^a^ | 9.5±0.1^a^ | 3527.1±106.1^a^ | 3574.9±106.0^a^ |
|  | C | 1985.3±33.5^b^ | 6.8±0.1^b^ | 2318.2±40.6^b^ | 2374.7±70.5^b^ |
| **Fungus** | CKT0 | 877.3±173.6^a^ | 6.2±0.5^a^ | 923.8±174.9^a^ | 943.2±167.1^a^ |
|  | CKT1 | 809.0±145.2^b^ | 6.1±0.4^a^ | 908.4±137.9^a^ | 923.8±150.1^a^ |
|  | C | 321.0±4.0^c^ | 3.4±0.1^b^ | 377.0±12.4^b^ | 400.7±8.4^b^ |

Soils amended with or without catalpol were incubated for 60 days at 26 ℃ at 50 % humidity. CKT0 and CKT1 represent control soil without catalpol and collected at experimental initiation and termination, respectively. C represents soil treated with catalpol for 60 days. Means are the average of three replicates.


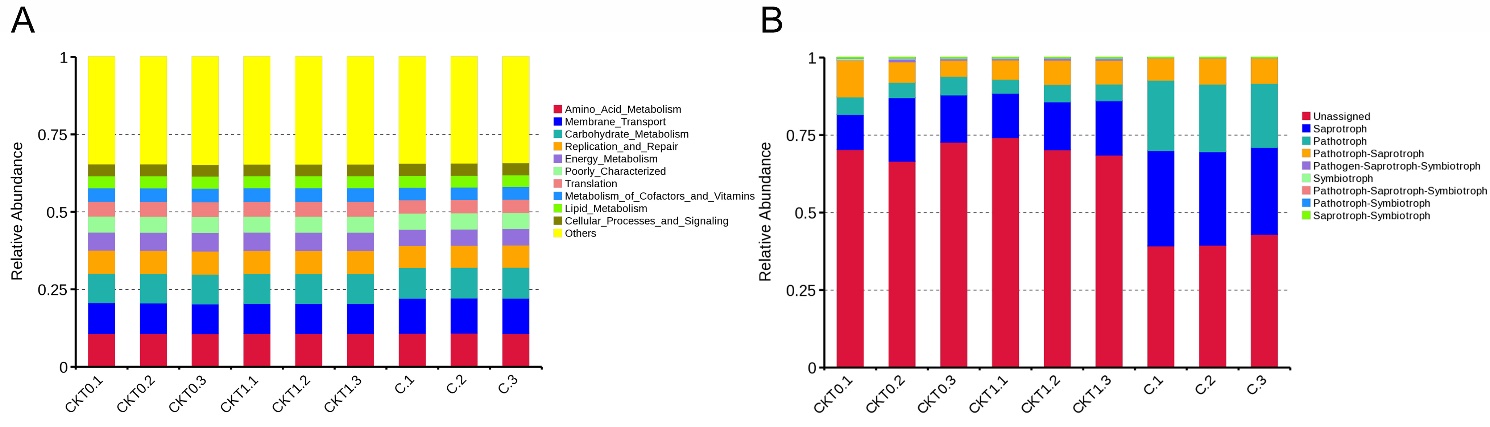


**Supplementary Figure S7.** Relative abundance of OTUs assigned to bacterial (A) and fungal (B) taxonomic groups. Soils amended with and without catalpol were incubated for 60 days at 26 ℃ at 50 % humidity. CKT0 and CKT1 represent control soil treatments without catalpol and collected at experimental initiation (T0) and termination (T1 at 60 days) of incubation, respectively. C represents treated soil amended with catalpol for 60 days and collected for analysis. Results represent the means of three replicates.


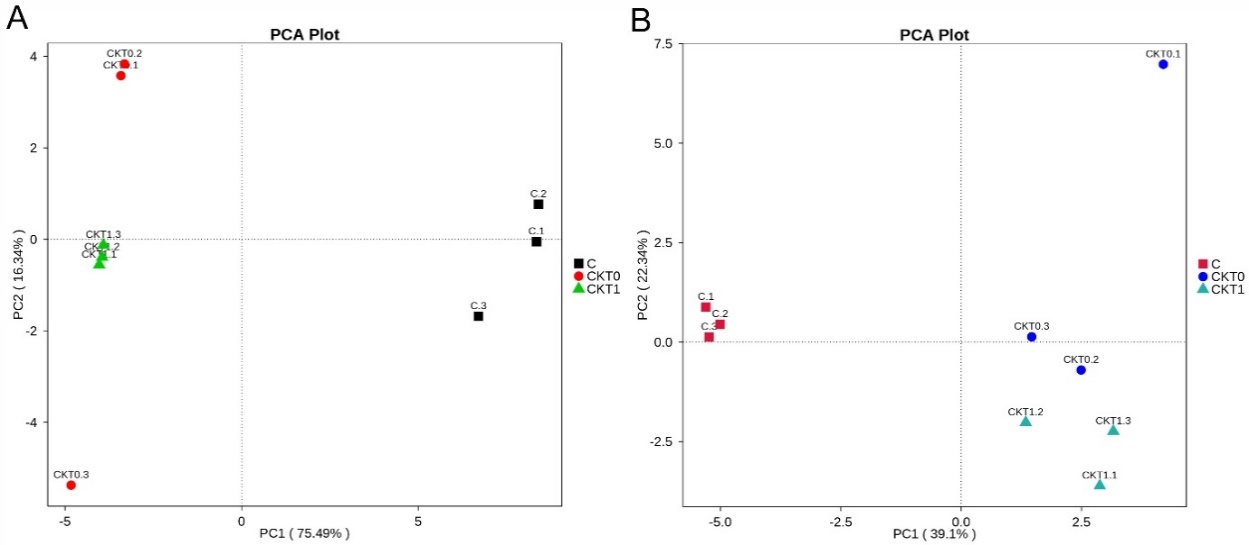


**Supplementary Figure S8.** Principal coordinate analysis (PCoA) of predicted bacterial (A) and fungal (B) functional groups. Soils amended with and without catalpol were incubated for 60 days at 26 ℃ at 50 % humidity. CKT0 and CKT1 represent control soil treatments without catalpol and collected at experimental initiation (T0) and termination (T1 at 60 days) of incubation, respectively. C represents treated soil amended with catalpol for 60 days and collected for analysis. Results represent the means of three replicates.


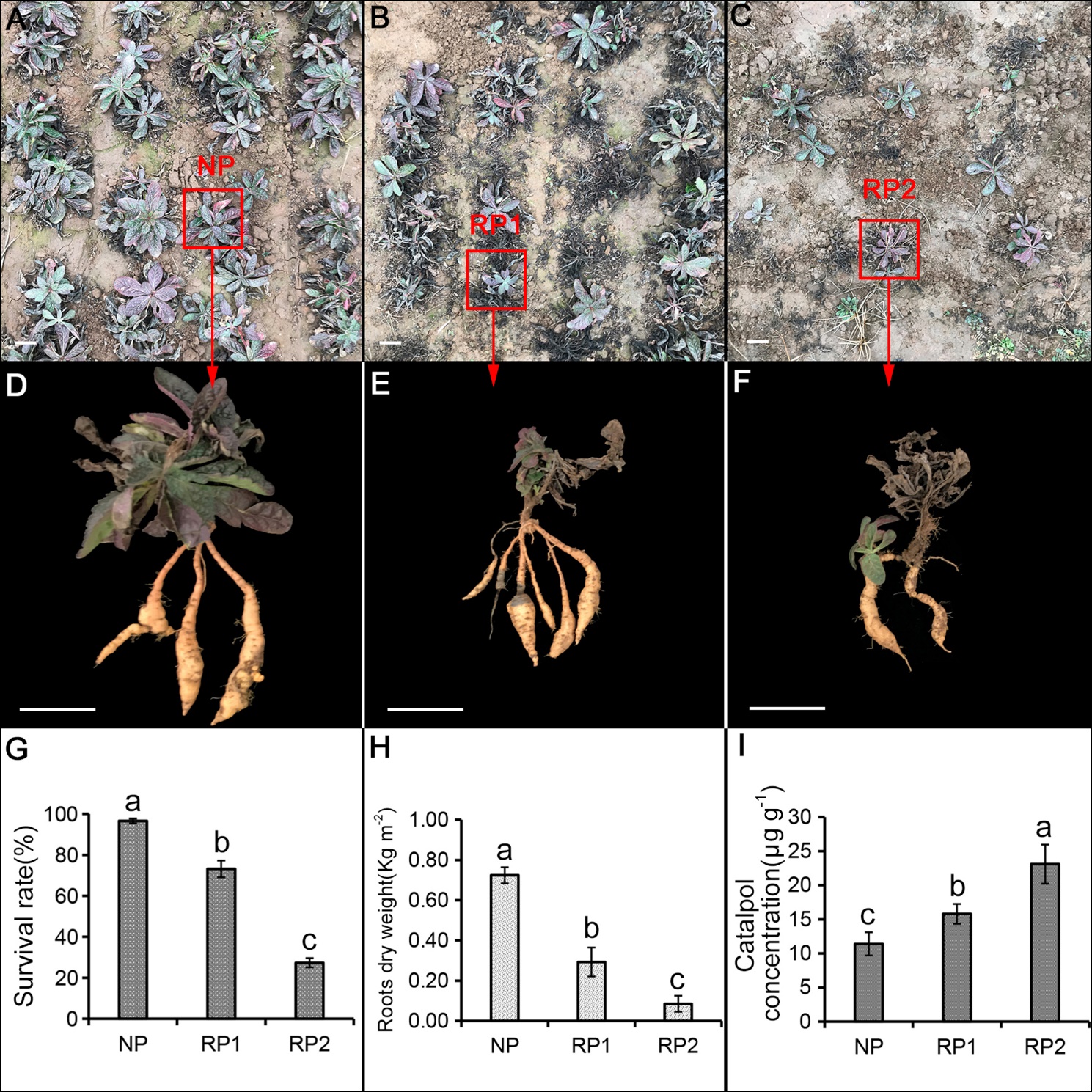


**Supplementary Figure S9.** Investigation of growth status of *R. glutinosa* and quantification of catalpol in the field replanted for different years. A: Newly planted (NP) *R. glutinosa*. Bar, 10 cm; B: Replanted *R. glutinosa* for one year (RP1). Bar, 10 cm; C: Replanted *R. glutinosa* for two years (RP2). Bar, 10 cm; D, E and F: Growth status of *R. glutinosa* under NP, RP1 and RP2 conditions, respectively. G: Survival rate of *R. glutinosa* under NP, RP1 and RP2 conditions. H: Roots dry weight of *R. glutinosa* under NP, RP1 and RP2 conditions. I: Quantification of catalpol in the rhizosphere soil of *R. glutinosa* under NP, RP1 and RP2 conditions.

**16S rRNA sequence of strain Rh-7 (*Pseudomonas aeruginosa*) (1325bp):**

GCGGCGGACGGGTGAGTAATGCCTAGGAATCTGCCTGGTAGTGGGGGATAACGTCCGGAAACGGGCGCTAATACCGCATACGTCCTGAGGGAGAAAGTGGGGGATCTTCGGACCTCACGCTATCAGATGAGCCTAGGTCGGATTAGCTAGTTGGTGGGGTAAAGGCCTACCAAGGCGACGATCCGTAACTGGTCTGAGAGGATGATCAGTCACACTGGAACTGAGACACGGTCCAGACTCCTACGGGAGGCAGCAGTGGGGAATATTGGACAATGGGCGAAAGCCTGATCCAGCCATGCCGCGTGTGTGAAGAAGGTCTTCGGATTGTAAAGCACTTTAAGTTGGGAGGAAGGGCAGTAAGTTAATACCTTGCTGTTTTGACGTTACCAACAGAATAAGCACCGGCTAACTTCGTGCCAGCAGCCGCGGTAATACGAAGGGTGCAAGCGTTAATCGGAATTACTGGGCGTAAAGCGCGCGTAGGTGGTTCAGCAAGTTGGATGTGAAATCCCCGGGCTCAACCTGGGAACTGCATCCAAAACTACTGAGCTAGAGTACGGTAGAGGGTGGTGGAATTTCCTGTGTAGCGGTGAAATGCGTAGATATAGGAAGGAACACCAGTGGCGAAGGCGACCACCTGGACTGATACTGACACTGAGGTGCGAAAGCGTGGGGAGCAAACAGGATTAGATACCCTGGTAGTCCACGCCGTAAACGATGTCGACTAGCCGTTGGGATCCTTGAGATCTTAGTGGCGCAGCTAACGCGATAAGTCGACCGCCTGGGGAGTACGGCCGCAAGGTTAAAACTCAAATGAATTGACGGGGGCCCGCACAAGCGGTGGAGCATGTGGTTTAATTCGAAGCAACGCGAAGAACCTTACCTGGCCTTGACATGCTGAGAACTTTCCAGAGATGGATTGGTGCCTTCGGGAACTCAGACACAGGTGCTGCATGGCTGTCGTCAGCTCGTGTCGTGAGATGTTGGGTTAAGTCCCGTAACGAGCGCAACCCTTGTCCTTAGTTACCAGCACCTCGGGTGGGCACTCTAAGGAGACTGCCGGTGACAAACCGGAGGAAGGTGGGGATGACGTCAAGTCATCATGGCCCTTACGGCCAGGGCTACACACGTGCTACAATGGTCGGTACAAAGGGTTGCCAAGCCGCGAGGTGGAGCTAATCCCATAAAACCGATCGTAGTCCGGATCGCAGTCTGCAACTCGACTGCGTGAAGTCGGAATCGCTAGTAATCGTGAATCAGAATGTCACGGTGAATACGTTCCCGGGCCTTGTACACACCGCCCGTCACACCATGGGAGTGGGTTG.

**ITS sequence of strain Rf-1 (*Fusarium oxysporum* CCS043) (573bp):**

CAAAATGCGGAAATGCAACGTGATCGAGGTCACATTCAGAAGTTGGGGGTTTAACGGCTTGGCCGCGCCGCGTTCCAGTTGCGAGGGTTTTACTACTACGCAATGGAGGCTGCAGCGAGACCGCCACTAGATTTCGGGGCCGGCTTGCCGCAAGGGCTCGCCGATCCCCAACACCAAACCCGAGGGCTTGAGGGTTGAAATGACGCTCGAACAGGCATGCCCGCCAGAATACTGGCGGGCGCAGTGTGCGTTCAAAGATTCTATGATTCACTGAATTCTGCAATTCACATTACTTATCGCATTTTGCTGCGTTCTTCATCGATGCCAGAACCGATAGATCCGTTGTTGAAAGTTTTGATTTATTTATGGGTTGACGCGGAAGTTACATATAGAAACAGAGTTTAGGGGCCCTCTGGAGGGGCGGCCCCTTTGCCCGCGCCCTTGCTGATCCGCCGACCCTCACAACTGGAACGCGGCGCGGCCAACCCGTTAAACCCCCAACTTCTGAATGTTGACCTCGGATCAGGTAGGAATACCCGCTGAACTTAAGCATATCAATAAGCGGCAGAATT

**ITS sequence of strain Rf-2 (*Fusarium solani*) (500bp):**

TGACATACCTATAACGTTGCCTCGGCGGGAACAGACGGCCCCGTAACACGGGCCGCCCCCGCCAGAGGACCCCCTAACTCTGTTTCTATAATGTTTCTTCTGAGTAAACAAGCAAATAAATTAAAACTTTCAACAACGGATCTCTTGGCTCTGGCATCGATGAAGAACGCAGCGAAATGCGATAAGTAATGTGAATTGCAGAATTCAGTGAATCATCGAATCTTTGAACGCACATTGCGCCCGCCAGTATTCTGGCGGGCATGCCTGTTCGAGCGTCATTACAACCCTCAGGCCCCCGGGCCTGGCGTTGGGGATCGGCGGAAGCCCCCTGCGGGCACAACGCCGTCCCCCAAATACAGTGGCGGTCCCGCCGCAGCTTCCATTGCGTAGTAGCTAACACCTCGCAACTGGAGAGCGGCGCGGCCACGCCGTAAAACACCCAACTTCTGAATGTTGACCTCGAATCAGGTAGGAATACCCGCTGAACTTAAGCATATCAA
